# Aortic Dissection Presenting as a STEMI

**DOI:** 10.21980/J8W647

**Published:** 2022-07-15

**Authors:** Jennifer Yee, Andrew P Kendle

**Affiliations:** * The Ohio State University, Department of Emergency Medicine, Columbus, OH

## Abstract

**Audience:**

This scenario was developed to educate emergency medicine residents on the presentation and management of a patient with a Stanford type A aortic dissection.

**Introduction:**

Chest pain is one of the most common chief complaints seen in the emergency department with a deadly differential diagnosis list. A “can’t miss” diagnosis, aortic dissection occurs when an intimal tear creates a false lumen in the aorta, with a variably reported incidence of approximately 2.5–5 per 100,000 person-years.^1^ This amounts to an estimated 8,000–16,000 cases per year in the United States with a mortality likely underestimated due to prehospital death ranging from 20–40% within 24 hours and 30–50% at 5 years.^2^,^3^,^4^ There is a reported increase in mortality by 1% for every hour the diagnosis is delayed, and half of diagnoses are made greater than 24 hours after presentation.^5^ The symptoms can range from chest pain to back pain, abdominal pain or extremity pain, to syncope or isolated neurologic deficits, even to shock or cardiac arrest.^6^ Aortic dissection is most commonly categorized into two groups: Stanford type A, involving the ascending aorta, and Stanford type B, involving only the descending aorta, and are generally managed surgically vs. medically respectively based on this paradigm.^7^,^8^ Stanford type A can be complicated by severe aortic regurgitation, pericardial tamponade or coronary artery occlusion mimicking ST-segment elevation myocardial infarction (STEMI). These potentials make it important to switch from heuristic to analytical thinking when developing a differential diagnosis.^9^ A high index of suspicion with early recognition and management is critical in this catastrophic disease state, especially given the propensity for complications and a wide variety of presentations.

**Educational Objectives:**

At the conclusion of the simulation session or during the debriefing session, learners will be able to: 1) Verbalize the anatomical differences and management of Stanford type A and type B aortic dissections, 2) Describe physical exam findings that may be found with ascending aortic dissections, 3) Describe the various clinical manifestations of the propagation of aortic dissections, 4) Discuss the management of aortic dissection, including treatment and disposition.

**Educational Methods:**

This session was conducted using a simulation scenario with a high-fidelity manikin as the patient and confederate/actor in the nursing role, followed by a post-scenario debriefing session on the presentation, differential diagnosis, potential physical exam findings, and management of patients with aortic dissection. Debriefing methods may be left to the discretion of the educators, but the authors have utilized advocacy-inquiry techniques.^10^ This scenario may also be run as an oral board examination case.

**Research Methods:**

The residents are provided an electronic survey at the completion of the debriefing session to anonymously rate different aspects of the simulation, as well as provide qualitative feedback on the scenario. This survey is specific to the local institution’s simulation center.

**Results:**

Twenty learners completed a feedback form. This session received all 6 and 7 scores (consistently effective/very good and extremely effective/outstanding, respectively) other than one isolated 5 score. The lowest average score was 6.5 for, “Before the simulation, the instructor set the stage for an engaging learning experience,” and the highest average score was 6.84 for, “The instructor identified what I did well or poorly - and why.” Feedback from the residents was overwhelmingly positive (available upon request). All groups initially gave aspirin upon identification of the STEMI and several gave heparin. Debriefing topics included STEMI mimics, physical exam findings for aortic dissection, imaging and laboratory workup for aortic dissection, blood pressure and heart rate goals and pharmacologic management, uncomplicated STEMI management, and Type I versus Type II decision-making.

**Discussion:**

This is an easily reproducible method for reviewing management of patients with aortic dissection. There are multiple potential presentations and complications of aortic dissections to further customize the experience for learners’ needs. While it was discussed during debriefing that heparin administration was unlikely to cause immediate cardiopulmonary arrest, this state was included to reflect downstream hemorrhagic complications that may occur in the setting of antiplatelet administration for acute aortic dissection. Facilitators may choose to omit the arrest at their discretion.

**Topics:**

Medical simulation, emergency medicine, aortic dissection, ST-elevation myocardial infarction, cardiovascular emergencies, hypertensive emergencies, STEMI mimics, vascular surgery, cardiothoracic surgery.

## USER GUIDE

[Table t1-jetem-7-3-s26]

**Table t1-jetem-7-3-s26:** 

**List of Resources:**	
Abstract	26
User Guide	28
Instructor Materials	30
Operator Materials	42
Debriefing and Evaluation Pearls	46
Simulation Assessment	50

**Learner Audience:**

Medical students, interns, junior residents, senior residents, emergency medical service (EMS) and first responders

**Time Required for Implementation:**

**Instructor Preparation:** 30 minutes

**Time for case:** 20 minutes

**Time for debriefing:** 40 minutes

**Recommended Number of Learners per Instructor:** 3–4

**Topics:**

Medical simulation, emergency medicine, aortic dissection, ST-elevation myocardial infarction, cardiovascular emergencies, hypertensive emergencies, STEMI mimics, vascular surgery, cardiothoracic surgery.

**Objectives:**

At the conclusion of this simulation, learners will be able to:

1. Differentiate between the anatomical differences and management of Stanford type A and type B aortic dissections
2. Demonstrate physical exam findings that may be found with ascending aortic dissections
3. Describe the various clinical manifestations of the propagation of aortic dissections
4. Discuss the management of aortic dissection, including treatment and disposition

### Linked objectives and methods

Aortic dissection is a critical, elusive and time-sensitive diagnosis in a patient presenting with chest pain. In this case, providers will review the following high-yield aspects of aortic dissections. Providers will learn features and anatomy of a Stanford type A dissection and contrast to a type B aortic dissection (Objective 1). This will include a review of historical and physical exam findings associated with aortic dissection (Objective 2), clinical manifestations and complications of aortic dissection propagation (Objective 3), and the management of aortic dissection (Objective 4). Objectives were tracked by facilitators taking notes during the simulation scenario for further discussion during debriefing.

This simulation scenario allows learners to reinforce their aortic dissection diagnosis and management skills in a physically and psychologically-safe learning environment, and then receive formative feedback on their performance.

### Recommended pre-reading for instructor

The authors recommend instructors review literature regarding the consensus treatment for aortic dissection, including epidemiology, presenting signs/symptoms, diagnosis, and management. Suggested readings include materials listed below under the “References/suggestions for further reading” section.

### Results and tips for successful implementation

This simulation was written to be performed as a high-fidelity simulation scenario, but also may be used as a mock oral board case.

The case was written for emergency medicine residents. This aortic dissection simulation case was conducted for approximately 30 emergency medicine residents during December 2021. The residents found the initial steps of management challenging, as all residents provided antiplatelets and several ordered heparin upon receipt of the initial electrocardiogram.

To make the case easier, the patient may emphasize different symptoms, such as upper back pain, the classically-taught “tearing” chest pain, simultaneous chest and abdominal pain, or divulge use of cocaine or a family history of connective tissue disorders. The previous chest x-ray may also be shown unprompted after learners review the new chest x-ray’s images.

An oral boards style-presentation may reveal the diagnosis earlier because a facilitator asking learners exactly what they are looking for in an organ system with a positive physical exam finding may reveal a murmur or asymmetrical pulses to palpation, regardless of the learner’s intent.

The local institution’s simulation center feedback form is based on the Center of Medical Simulation’s Debriefing Assessment for Simulation in Healthcare (DASH) Student Version Short Form^11^ with the inclusion of required qualitative feedback if an element was scored less than a 6 or 7. Twenty learners completed a feedback form. This session received all 6 and 7 scores (consistently effective/very good and extremely effective/outstanding, respectively) other than one isolated 5 score. The lowest average score was 6.5 for “Before the simulation, the instructor set the stage for an engaging learning experience,” and the highest average score was 6.84 for “The instructor identified what I did well or poorly - and why.” The form also includes an area for general feedback about the case at the end. Illustrative examples of feedback include: “Really enjoy these, always great refreshers and question-provoking,” and “Great case with a lot of learning.”

## Supplementary Information





































## Figures and Tables

**Table t2-jetem-7-3-s26:** 

#1	Inferior STEMI electrocardiogram (ECG)
#2	Previous ECG
#3	Portable chest radiograph (CXR) with widened mediastinum
#4	Previous CXR accessed by Picture Archiving and Communication System (PACS)
#5	Complete blood count (CBC)
#6	Basic metabolic panel (BMP)
#7	Liver function tests
#8	Lipase
#9	Calcium
#10	Magnesium
#11	Phosphorous
#12	Troponin, low-sensitivity
#13	Troponin, high-sensitivity
#14	D-dimer
#15	Coagulation factors
#16	Brain natriuretic peptide (BNP)
#17	Computed tomography (CT) angiography (CTA) of the chest

**Table t3-jetem-7-3-s26:** 

**Vitals:**	Heart Rate (HR) 115	Blood Pressure (BP) 190/90 right arm
	Respiratory Rate (RR)	24 Temperature (T) 98°F
	Oxygen Saturation (O_2_ Sat) 98% on room air	

**Figure f1-jetem-7-3-s26:**
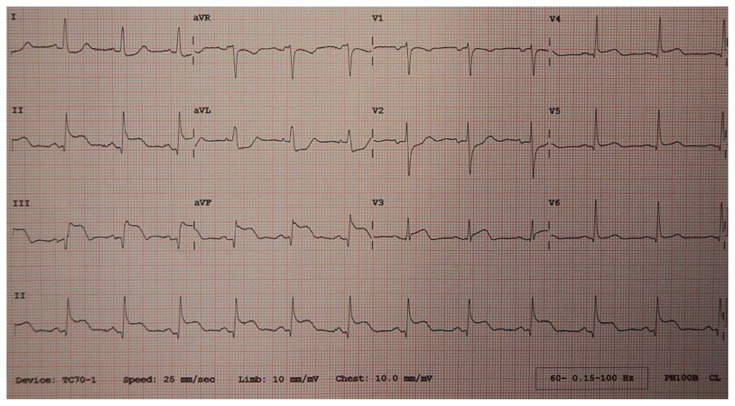


**Figure f2-jetem-7-3-s26:**
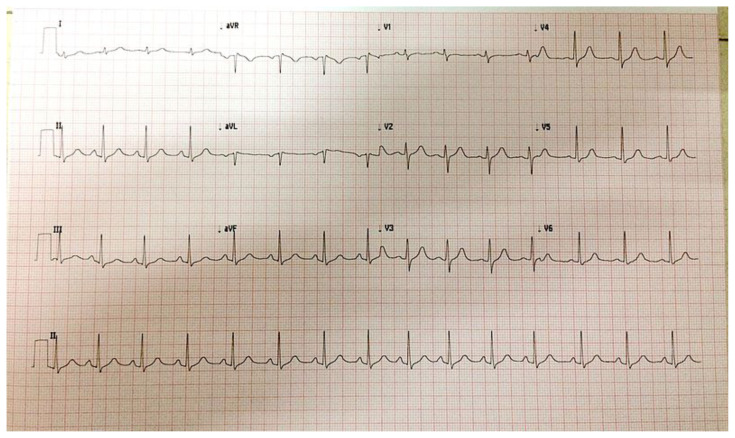


**Figure f3-jetem-7-3-s26:**
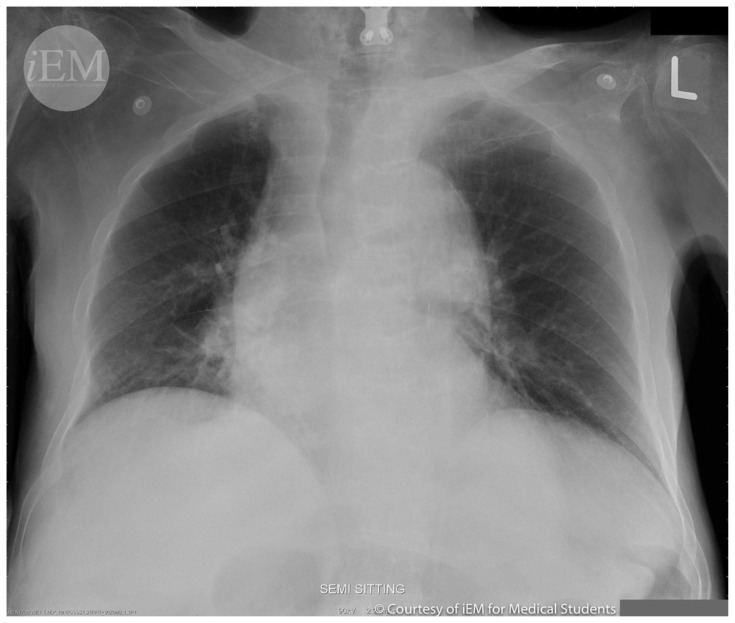


**Figure f4-jetem-7-3-s26:**
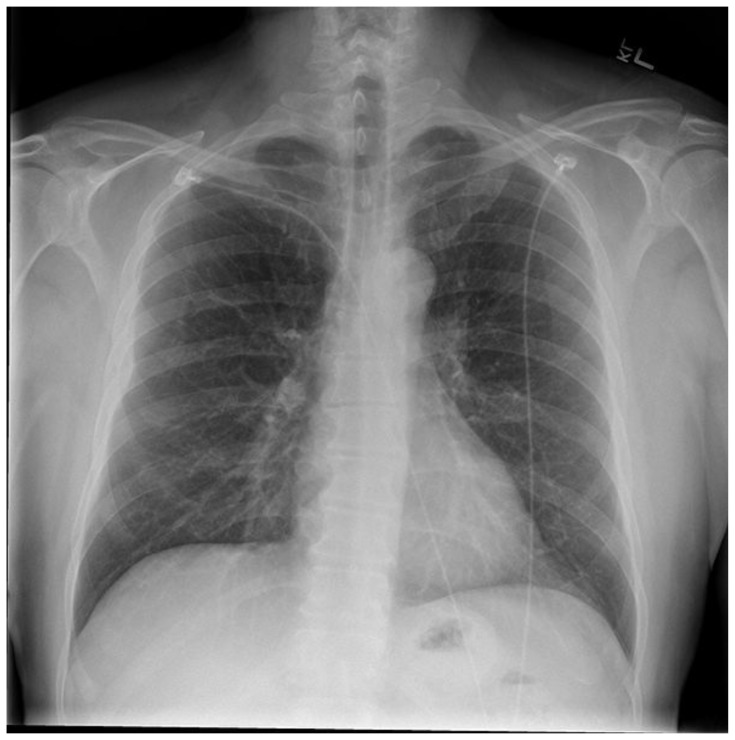


**Table t4-jetem-7-3-s26:** 

*Complete blood count (CBC)*	
White blood count (WBC)	14.2 **×** 1000/mm^3^
Hemoglobin (Hgb)	13.5 g/dL
Hematocrit (HCT)	40.5%
Platelet (Plt)	180 **×** 1000/mm^3^

**Table t5-jetem-7-3-s26:** 

*Basic metabolic panel (BMP)*	
Sodium	133 mEq/L
Potassium	4.2 mEq/L
Chloride	99 mEq/L
Bicarbonate (HCO_3_)	25 mEq/L
Blood Urea Nitrogen (BUN)	38 mg/dL
Creatinine (Cr)	1.1 mg/dL
Glucose	100 mg/dL
Calcium	8.0 mg/dL

**Table t6-jetem-7-3-s26:** 

*Liver function test (LFTs)*	
Total bilirubin	1.4 mg/dL
Direct bilirubin	0.2 mg/dL
Alkaline phosphatase	90 units/L
Aspartate aminotransferase (AST)	39 units/L
Alanine aminotransferase (ALT)	13 units/L
Albumin	4.1 g/dL
*Lipase*	155 units/L
*Magnesium*	1.3 mEq/L
*Phosphorous*	3.5 mg/dL
*Troponin, low-sensitivity*	0.09 ng/mL (<0.03 ng/mL)
*Troponin, high-sensitivity*	90 ng/L (<33 ng/L)
*D-dimer*	1.4 ug/mL (<0.5 ug/mL)

**Table t7-jetem-7-3-s26:** 

*Coagulation Panel*	
Prothrombin time (PT)	20 seconds
Partial thromboplastin time (PTT)	65 seconds
INR	1.7
*BNP*	20 pg/mL

**Figure f5-jetem-7-3-s26:**
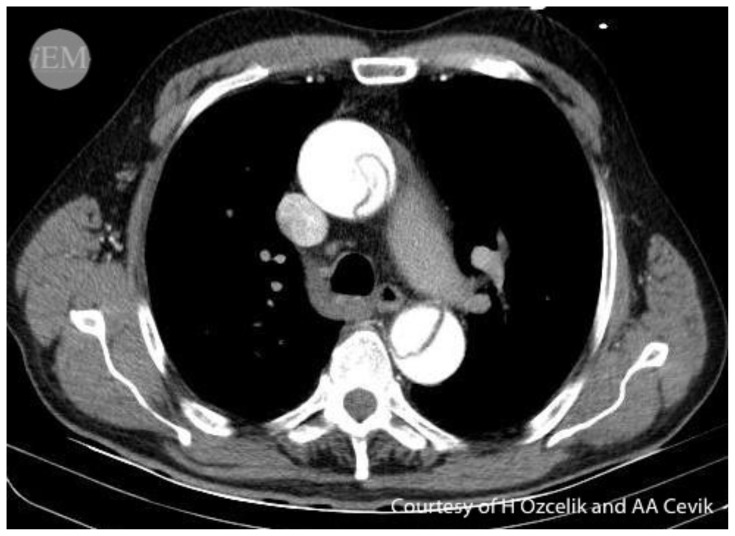


**Table:** SIMULATION EVENTS TABLE:

Minute (state)	Participant action/trigger	Patient status (simulator response) & operator prompts	Monitor display (vital signs)
0:00 (Baseline)	Learners are brought into the simulation bay and given an ECG by nursing.	Learners should begin by interpreting the ECG, reviewing initial vitals, and assessing the patient through a focused history and exam.	T 98°F HR 115 BP 190/90 right upper extremity (RUE) BP 230/120 left upper extremity (LUE) if checked RR 24 O2 98% RA
4:00	Labs, chest x-ray, and/or CT angiography should be ordered. Patient should be given analgesia.	Patient has continued chest pain. If a STEMI alert is activated or if the cath lab/cardiologist is contacted, learners will be notified that the cardiologist is in the cath lab with a patient and is unable to return pages for the next 30 minutes.	T 98°F HR 115 BP 190/90 RUE BP 230/120 LUE if checked RR 24 O2 98% RA
6:00	Hypertension and tachycardia will worsen at minute 6.	If learners order IV push medications to decrease heart rate/blood pressure, vital signs will respond as below: 10mg labetalol or 10mg hydralazine push: BP decreases to 170/80 right arm 5mg metoprolol push: heart rate decreases to 90 bpm If learners order titratable medications to decrease heart rate/blood pressure, nursing will ask what their vital sign parameter goals are and vital signs will respond appropriately to stated goal over 1 minute.	T 98°F HR 130 BP 210/120 RUE BP 244/130 LUE if checked RR 24 O2 98% RA 10mg Labetalol or hydralazine only: T 98°F HR 118 BP 170/80 RUE BP 190/90 LUE if checked RR 24 O2 98% RA 5mg Metoprolol only: T 98°F HR 94 BP 200/110 RUE BP 234/128 LUE if checked RR 24 O2 98% RA Titratable medications ordered: T 98°F HR to stated goal over 1 minute BP to stated goal over 1 minute RR 24 O2 98% RA
10:00 (Case Completion)	Learners voice concern for aortic dissection and contact cardiothoracic surgery.	If heparin had been given, patient then goes into PEA arrest (sinus tachycardia at 160 beats per minute) with return of spontaneous circulation occurring per facilitator’s discretion. Alternatively: An angry CT surgeon calls in prior to PEA arrest and admonishes team for administering antiplatelets and/or heparin, but ultimately accepts the patient and takes her to the OR. If heparin has not been given, cardiothoracic surgery asks what their goal vital sign parameters are and accepts the patient to be taken to the operating room.	Heparin given: T 98°F HR 160 (PEA) BP undetectable RR undetectable O2 undetectable Untreated vitals but no heparin given: T 98°F HR 130 BP 210/120 RUE BP 244/130 LUE if checked RR 24 O2 98% RA Labetalol or hydralazine only but no heparin given: T 98°F HR 118 BP 170/80 RUE BP 190/90 LUE if checked RR 24 O2 98% RA Metoprolol only but no heparin given: T 98°F HR 94 BP 200/110 RUE BP 234/128 LUE if checked RR 24 O2 98% RA Titratable medications ordered but no heparin given: T 98°F HR to stated goal over 1 minute BP to stated goal over 1 minute RR 24 O2 98% RA

**Table:** 

**Critical Actions:** Recognition of diastolic murmur Palpate radial pulses simultaneously Correctly interpreting CXR as having a widened mediastinum Ordering beta-blockade to decrease heart rate to a goal of 60 beats per minute Ordering anti-hypertensive therapy to a goal systolic blood pressure of 100–120 mmHg Contacting cardiothoracic surgery for disposition	0:00

**Table:** 

	Milestone	Did not achieve level 1	Level 1	Level 2	Level 3
1	**Emergency Stabilization (PC1)**	□ Did not achieve Level 1	□ Recognizes abnormal vital signs	□ Recognizes an unstable patient, requiring intervention Performs primary assessment Discerns data to formulate a diagnostic impression/plan	□ Manages and prioritizes critical actions in a critically ill patient Reassesses after implementing a stabilizing intervention
2	**Performance of focused history and physical (PC2)**	□ Did not achieve Level 1	□ Performs a reliable, comprehensive history and physical exam	□ Performs and communicates a focused history and physical exam based on chief complaint and urgent issues	□ Prioritizes essential components of history and physical exam given dynamic circumstances
3	**Diagnostic studies (PC3)**	□ Did not achieve Level 1	□ Determines the necessity of diagnostic studies	□ Orders appropriate diagnostic studies. Performs appropriate bedside diagnostic studies/procedures	□ Prioritizes essential testing Interprets results of diagnostic studies Reviews risks, benefits, contraindications, and alternatives to a diagnostic study or procedure
4	**Diagnosis (PC4)**	□ Did not achieve Level 1	□ Considers a list of potential diagnoses	□ Considers an appropriate list of potential diagnosis May or may not make correct diagnosis	□ Makes the appropriate diagnosis Considers other potential diagnoses, avoiding premature closure
5	**Pharmacotherapy (PC5)**	□ Did not achieve Level 1	□ Asks patient for drug allergies	□ Selects an medication for therapeutic intervention, consider potential adverse effects	□ Selects the most appropriate medication and understands mechanism of action, effect, and potential side effects Considers and recognizes drug-drug interactions
6	**Observation and reassessment (PC6)**	□ Did not achieve Level 1	□ Reevaluates patient at least one time during case	□ Reevaluates patient after most therapeutic interventions	□ Consistently evaluates the effectiveness of therapies at appropriate intervals
7	**Disposition (PC7)**	□ Did not achieve Level 1	□ Appropriately selects whether to admit or discharge the patient	□ Appropriately selects whether to admit or discharge Involves the expertise of some of the appropriate specialists	□ Educates the patient appropriately about their disposition Assigns patient to an appropriate level of care (ICU/Tele/Floor) Involves expertise of all appropriate specialists
9	**General Approach to Procedures (PC9)**	□ Did not achieve Level 1	□ Identifies pertinent anatomy and physiology for a procedure Uses appropriate Universal Precautions	□ Obtains informed consent Knows indications, contraindications, anatomic landmarks, equipment, anesthetic and procedural technique, and potential complications for common ED procedures	□ Determines a back-up strategy if initial attempts are unsuccessful Correctly interprets results of diagnostic procedure
20	**Professional Values (PROF1)**	□ Did not achieve Level 1	□ Demonstrates caring, honest behavior	□ Exhibits compassion, respect, sensitivity and responsiveness	□ Develops alternative care plans when patients’ personal beliefs and decisions preclude standard care
22	**Patient centered communication (ICS1)**	□ Did not achieve level 1	□ Establishes rapport and demonstrates empathy to patient (and family) Listens effectively	□ Elicits patient’s reason for seeking health care	□ Manages patient expectations in a manner that minimizes potential for stress, conflict, and misunderstanding. Effectively communicates with vulnerable populations, (at risk patients and families)
23	**Team management (ICS2)**	□ Did not achieve level 1	□ Recognizes other members of the patient care team during case (nurse, techs)	□ Communicates pertinent information to other healthcare colleagues	□ Communicates a clear, succinct, and appropriate handoff with specialists and other colleagues Communicates effectively with ancillary staff

## INSTRUCTOR MATERIALS

**Case 1 Title:** Aortic Dissection Presenting as a STEMI

**Case Description & Diagnosis (short synopsis):** Patient is a 50-year-old female who presents to an academic emergency department (“American Board of Emergency Medicine General”) with chest pain. The confederate/actor in the nursing role will start the case by first presenting an electrocardiogram (ECG) to the learners, stating, “50-year-old female with chest pain came into triage. Here’s her ECG. We just brought her back into a bed.” If nursing is asked, they urgently brought her into a bed because “we didn’t like how her ECG looked.” Her chest pain is sharp, radiates to her back, began suddenly, and causes her difficulty in breathing, but she will not divulge radiation to the back unless specifically asked. The patient is diaphoretic with discrepant radial pulses to palpation and an aortic regurgitation murmur. If the manikin is unable to demonstrate these physical exam changes, they can be verbalized by the nurse or by the facilitator if learners specifically ask if these findings are present, or if they simultaneously check bilateral radial pulses. If bilateral upper extremity blood pressures are obtained, there is a difference greater than 20 mmHg between both arms. Laboratory findings demonstrate an elevated troponin, an elevated d-dimer, and a chest x-ray (CXR) with a widened mediastinum. Hypertension and tachycardia will worsen at minute 6 but are amendable to appropriate medical therapies. Pain improves with opiate analgesia. If the patient is given heparin, the patient will undergo pulseless electrical activity (PEA) arrest and the case will end at the discretion of the facilitator. Alternatively, facilitators may have a cardiothoracic surgeon call in once the aortic dissection is identified and express their displeasure at heparin and/or antiplatelet medications having been administered.

If learners call an ST-elevation myocardial infarction (STEMI) alert or attempt to call cardiology/the catheterization (cath) lab, learners will be notified that cardiology is in the cath lab currently with another patient and are unable to return their page. If learners contact cardiothoracic surgery and have not given the patient heparin, the surgeon will be amenable to coming down to the emergency department and the case will end.

**Equipment or Props Needed:**

- One actor or confederate for the nursing role
- A high-fidelity manikin – the authors used a SimMan® **3G Manikin**
- An automated external defibrillator (AED) device or hospital-grade defibrillator
- A crash cart with advanced cardiovascular life support (ACLS) medications and airway equipment
- Moulage to apply diaphoresis (glycerin spray or water-soluble jelly with water sprayed upon the face)
- Cardiac monitor
- Pulse oximetry
- Peripheral intravenous (IV) line
- IV pole
- Simulated medications with labeling (not limited to: labetalol, hydralazine, metoprolol, esmolol, nitroprusside, nicardipine, morphine, nitroglycerin, fentanyl, aspirin, ticagrelor, clopidogrel, heparin)
- Intravenous fluids, including normal saline and lactated ringers
- Intravenous fluid pressure bag

**Confederates needed:**

One actor or confederate in the nursing role

**Stimulus Inventory:** [Table t2-jetem-7-3-s26]

**Background and brief information:** Patient presents to triage in an academic emergency department with sharp chest pain. The nurse presents the ECG to the learners and then brings them to the patient’s bedside for evaluation.

**Initial presentation:** Patient is Donna Prince, a 50-year-old female who presented to triage with sharp chest pain.

- Past medical history: hypertension (HTN), hyperlipidemia
- Past surgical history: hysterectomy
- Medications: hydrochlorothiazide
- Allergies: no known drug allergies
- Family history: HTN, type-II diabetes mellitus, coronary artery disease (both parents)
- Vital signs:
  ○ Heart Rate (HR) – 115
  ○ Blood Pressure (BP) – 190/90 right upper extremity (230/120 left upper extremity – must specifically request for this to be performed)
  ○ Respiratory rate – 24
  ○ Oxygen saturation: 98% room air
  ○ Temperature – 98° F
- Weight: 170 lbs, 77 kg

Assessment: Patient is diaphoretic and mildly dyspneic. Repeatedly voices that she is in pain every 2 minutes.

**How the scene unfolds:** Patient is a 50-year-old female who presents to an academic emergency department with chest pain. The actor/confederate in the nursing role will start the case by first presenting an electrocardiogram (ECG) to the learners, stating “50-year-old female with chest pain came into triage. Here’s her ECG. We just brought her back into a bed.” If asked, a previous ECG is available. After the first ECG from today is reviewed, any repeat ECGs during this encounter will be unchanged. If the patient is asked specific details about her symptoms, her chest pain is sharp, radiates to her back, began suddenly, and causes her difficulty in breathing. The patient is diaphoretic with discrepant radial pulses to palpation and an aortic regurgitation murmur. If bilateral upper extremity blood pressures are checked, there is a difference greater than 20 mmHg between both arms. Laboratory findings demonstrate an elevated troponin, an elevated d-dimer, and a CXR with a widened mediastinum. If requested, a previous CXR will be shown that shows a normal mediastinum and a previous ECG will be shown that is non-ischemic. Any repeat ECGs after the first ECG will be unchanged. Hypertension and tachycardia will worsen at minute 6 but are amendable to appropriate medical therapies. If the patient is given heparin, the patient will undergo pulseless electrical activity (PEA) arrest and the case will end at the discretion of the facilitators, or, alternatively, cardiothoracic surgery calls in when the aortic dissection is diagnosed to admonish the team for giving the patient heparin and/or antiplatelet therapy.

If learners call an ST-elevation myocardial infarction (STEMI) alert or attempt to call cardiology/the catheterization (cath) lab, learners will be notified that cardiology is in the cath lab currently with another patient and are unable to return their page. If learners contact cardiothoracic surgery prior to the patient undergoing PEA arrest, the surgeon will be amenable to coming down to the emergency department and the case will end. Providing a hand-off to cardiothoracic surgery is strongly suggested as a required step prior to ending the case scenario. The length for all of these measures to be achieved should be approximately 15 minutes for a 3- to 4-person team.

Non-ideal management should include anti-platelet or heparin administration, not treating the patient’s pain, failure to perform a deliberate physical exam including checking for pulse symmetry and a cardiac murmur, failure to correctly interpret the patient’s CXR, failure to administer medications to lower the patient’s blood pressure and heart rate in a timely fashion, and delay of contacting cardiothoracic surgery.

**Critical actions:**

1. Recognition of diastolic murmur
2. Palpate radial pulses simultaneously (or obtain blood pressures in bilateral upper extremities)
3. Correctly interpreting CXR as having a widened mediastinum
4. Ordering beta-blockade to decrease heart rate to a goal of 60 beats per minute
5. Ordering anti-hypertensive therapy to a goal systolic blood pressure of 100–120 mmHg
6. Contacting cardiothoracic surgery for disposition

**Case Title:** Aortic Dissection Presenting as a STEMI

**Chief Complaint:** Patient is Donna Prince, a 50-year-old female who presents to triage in an academic emergency department with sharp chest pain.[Table t3-jetem-7-3-s26]

**General Appearance:** Diaphoretic, anxious. Well-groomed and well-dressed

**Primary Survey:**

- **Airway:** intact
- **Breathing:** equal breath sounds bilaterally, dyspneic at rest
- **Circulation:** heart regular tachycardic rhythm. 2+ left radial pulse, 1+ right radial pulse. Capillary refill <3 seconds.

**History:**

- **History of present illness:** Patient is Donna Prince, a 50-year-old female who presents to triage in an academic emergency department with sharp chest pain.
  ○ IF ASKED:
    - She has never had this pain before. Never had a stress test. No recent illness.
    - Pain began suddenly at rest thirty minutes ago while sitting at a desk.
    - She has associated presyncope, nausea, dyspnea, and back pain.
    - The pain is severe.
    - It is not pleuritic.
    - She did not try any medications to relieve the pain.
    - No history of smoking. No history of thromboembolic disease, recent travel or surgery, hemoptysis, estrogen use, or family history of clotting disorders.
- **Past medical history**: HTN, hyperlipidemia
- **Past surgical history:** hysterectomy
- **Medications:** hydrochlorothiazide
- **Allergies:** No known drug allergies
- **Social history:** No history of smoking
- **Family history:** HTN, type-II diabetes mellitus, coronary artery disease (both parents)

**Secondary Survey/Physical Examination:**

- **Vital signs**:
  ○ HR – 115
  ○ BP – 190/90 right upper extremity
  ○ BP – 230/120 left upper extremity
  ○ Resp rate – 24
  ○ Oxygen saturation 98% room air
  ○ Temp – 98°F
  ○ **Weight:** 170 lbs, 77 kg
- **General appearance:** Diaphoretic, in pain
- **HEENT:**
  ○ **Head:** diaphoretic, otherwise within normal limits (wnl)
  ○ **Eyes:** wnl. Pupillary exam 3mm with pupils equal and reactive to light bilaterally
  ○ **Ears:** wnl
  ○ **Nose:** wnl
  ○ **Throat:** wnl
- **Neck:** wnl
- **Heart:** regular tachycardic rhythm. A high pitched diastolic decrescendo murmur is present, 2+ left radial pulse, 1+ right radial pulse. Capillary refill <3 seconds. No lower extremity edema.
- **Lungs:** equal breath sounds bilaterally. Mildly dyspneic at rest as verbalized by the simulationist
- **Abdominal/GI:** wnl
- **Genitourinary:** deferred
- **Rectal:** deferred
- **Extremities:** 2+ left radial pulse, 1+ right radial pulse. Otherwise wnl
- **Back:** wnl
- **Neuro:** wnl
- **Skin:** diaphoretic, otherwise wnl
- **Lymph:** wnl
- **Psych:** wnl

*Inferior STEMI ECG*

Heilman, J. Inferior and RtV MI 12 lead. 12 lead ECG showing inferior and right ventricular infarct. File:Inferior and RtV MI 12 lead.jpg - Wikimedia Commons. Published 19 May 2014. CC BY-SA 4.0.

[Fig f1-jetem-7-3-s26] *Previous ECG*

. 10sec-ekg-lead. : 10 . In: Wikimedia Commons. https://commons.wikimedia.org/wiki/File:10sec-ekg-lead.jpg. Published 7 March 2017. CC BY-SA 4.0.[Fig f2-jetem-7-3-s26]

*Portable CXR with widened mediastinum*

iEM for Medical Students. 469 - wide mediastinum - chest X-ray. In: Wordpress. https://wordpress.org/openverse/image/46e4a786-b4f1-4b92-b9be-0079506df27d. CC BY-NC-SA 2.0.[Fig f3-jetem-7-3-s26]

*Previous CXR*

Chest x-ray – posteroanterior view

O’Dea. Chest x-ray – posteroanterior view. Mild diaphragmatic flattening may be indicative of chronic obstructive pulmonary disease. Lungs clear. No pneumothorax or effusion. Heart and pulmonary vasculature unremarkable. No acute disease. (ECG leads visible.) File:Chest x-ray - posteroanterior view.jpg - Wikimedia Commons. Published 13 June 2012. CC BY-SA 3.0.[Fig f4-jetem-7-3-s26]

[Table t4-jetem-7-3-s26] [Table t5-jetem-7-3-s26] [Table t6-jetem-7-3-s26] [Table t7-jetem-7-3-s26] *CTA Chest*

H Ozcelik and AA Cevik. 68.2 - Aortic Dissection. In: Wordpress. https://wordpress.org/openverse/image/bffc13e0-bf32-4a77-ab32-3285699b6335. CC BY-NC-SA 2.0.[Fig f5-jetem-7-3-s26]

## OPERATOR MATERIALS

**Diagnosis:**

Stanford Type-A aortic dissection complicated by an inferior STEMI

**Disposition:**

Operating room with cardiothoracic surgery

## DEBRIEFING AND EVALUATION PEARLS

### Aortic Dissection Presenting as a STEMI

**Learning Points:**

1. **ST segment elevation may be due to multiple different etiologies, some emergent and life-threatening. While diagnosing STEMI in a timely fashion is critical, it should not be a reflexive treatment pathway.**
2. **Aortic dissection is associated with genetic, family, social, and surgical risk factors.**
3. **Concerning historical elements include rapid onset of severe pain, sharp or tearing quality, radiating or migrating pain.**
4. **Exam findings in aortic dissection include pulse deficits or asymmetry, neuro deficits, aortic insufficiency murmur, or shock.**
5. **Complications of aortic dissection include ischemic limb, renal or abdominal vascular injury, stroke, STEMI, and tamponade**
6. **Due to the rate of delayed or missed diagnosis, a high index of suspicion is required.**

**Pearls:**

Aortic dissection risk factors: HTN, cocaine use, pregnancy, collagen disorders (Marfan, Ehlers-Danlos), bicuspid aortic valve, vasculitis.

In one study: Sixty-four percent describe sharp pain, and 50% describe tearing or ripping pain. 9.4% presented with syncope, which is more common in type A.^1^

Beware of those who:

- report chest AND abdominal pain (pain that crosses the diaphragm).
- report chest pain and neurologic symptoms.

Exam: know what you are looking for BEFORE you start your exam! (be deliberate)

- Check for arm BP differential >20 mmHg (left>right), but know that other patients without dissection may also have this.
- Aortic regurgitation (diastolic murmur), neurologic deficits, rales/jugular venous distention/hepatojugular reflex.^2^
- May be hypertensive or hypotensive (hypotension increases the diagnostic likelihood^3^ and worsens prognosis^4^).

D-dimer

- do not rely on D-dimer alone to exclude the diagnosis of aortic dissection.
- Low or false-negative D-dimer may be seen in:
  ○ Chronicity, time of symptom onset, presence of thrombosed false lumen, intramural hematoma, short length of dissection, young age.^5^

ECG:

- Patients can present as inferior STEMIs for two reasons.
  ○ The first is a dissection is more likely to occur in the greater curvature and thus more likely to obstruct the right coronary artery = right-sided heart failure symptoms.
  ○ Second, dissection into the left main is often fatal.^6^

CXR

- widening of the aortic silhouette >8 cm on a posterior-anterior film, pleural effusion left greater than right, right tracheal displacement, apical pleural cap, left paraspinal stripe, aortic kinking.^7^

Treatment:

- The ideal target blood pressure for acute aortic dissection management is currently undefined by controlled trials and must be tailored to each patient, but an appropriate goal is a systolic pressure of < 120 Hg while maintaining end-organ perfusion
  ○ A suggested heart rate goal is 60 beats per minute^8^
- Beta-1 selective adrenergic antagonists will decrease inotropy and chronotropy: esmolol, metoprolol
- Alpha-1:beta-1 and beta-2 adrenergic antagonist (7:1 alpha to beta ratio): intravenous labetalol
- Peripheral vasodilation: nitroprusside.
  ○ beta-1 blockade should be performed before initiating nitroprusside to prevent reflex tachycardia

Consequences of aortic dissection

- Stroke, tamponade, STEMI, end organ damage to kidneys with distal propagation of dissection
- Aneurysmal dilation of the aorta may compress regional structures such as the esophagus, the recurrent laryngeal nerve, or the superior cervical sympathetic ganglion, causing dysphagia, hoarseness, or Horner’s syndrome.

In one 4-year review, 39% (26/66) patients with acute aortic syndrome were misdiagnosed.^9^

- Increasing age and anterior chest pain predicted initial misdiagnosis
- Only 6% had definite coronary involvement
- 31% misdiagnosed patients did not have chest x-rays initially performed
  ○ Of those who did, 67% had mediastinal widening or abnormal aortic contours
  ○ Most with initial misdiagnosis had normal ECG and biomarkers
- Those who received antithrombotics have higher rates of major bleeding and a trend towards greater in-hospital mortality with a trend towards aortic rupture
  ○ Aspirin and clopidogrel use increased bleeding risk after cardiovascular surgery for days

Take aways:

- Do not order aspirin or heparin for patients with chest pain prior to evaluating them!
- Be deliberate in your exam
- Be deliberate in your decision making
  ○ Does something not fit the presumed diagnosis?
  ○ Could something else be causing this presentation?
  ○ Could something else be going on simultaneously?

**Other debriefing points:**

Closed-loop communication among team: Was it used? Why or why not? Were there any implications of this during case execution?

## SIMULATION ASSESSMENT

*Case: Aortic Dissection Presenting as a STEMI*

Learner: _________________________________________

***Assessment Timeline***

This timeline is to help observers assess their learners. It allows observer to make notes on when learners performed various tasks, which can help guide debriefing discussion.

**Critical Actions:**

□ Recognition of diastolic murmur
□ Palpate radial pulses simultaneously
□ Correctly interpreting CXR as having a widened mediastinum
□ Ordering beta-blockade to decrease heart rate to a goal of 60 beats per minute
□ Ordering anti-hypertensive therapy to a goal systolic blood pressure of 100–120 mmHg
□ Contacting cardiothoracic surgery for disposition

**Summative and formative comments:**

**Milestones assessment:**
