# Supplementary material for: Aortic Dissection Presenting as a STEMI
Source: J Educ Teach Emerg Med. 2022 Jul 15;7(3):S26–54. doi: 10.21980/J8W647 (PMC10332695; doi:10.21980/J8W647)
Supplement: Supplementary file 1 [file jetem-7-3-s26-supp1.pptx]

## Slide 1
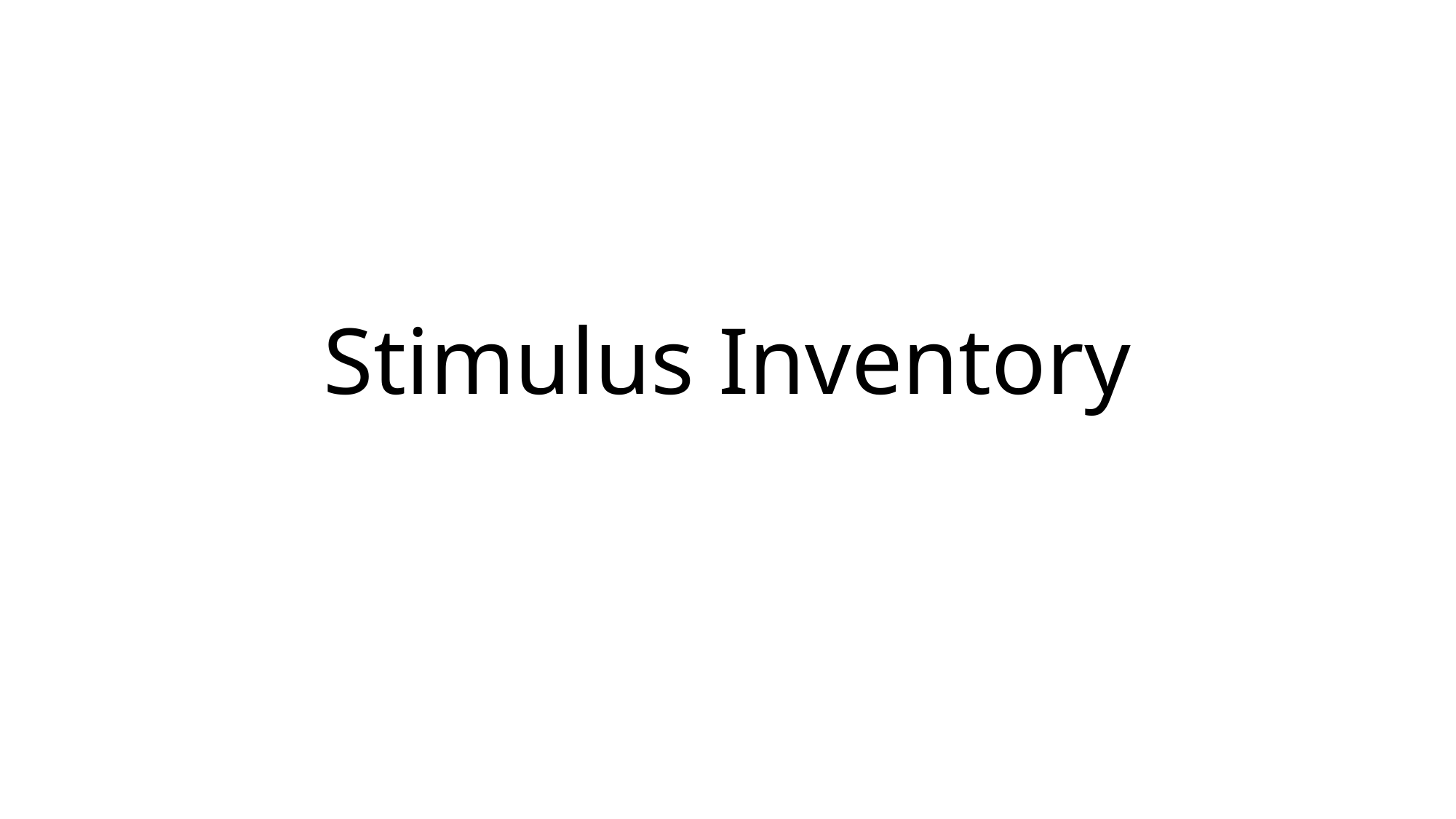

# Stimulus Inventory

## Slide 2
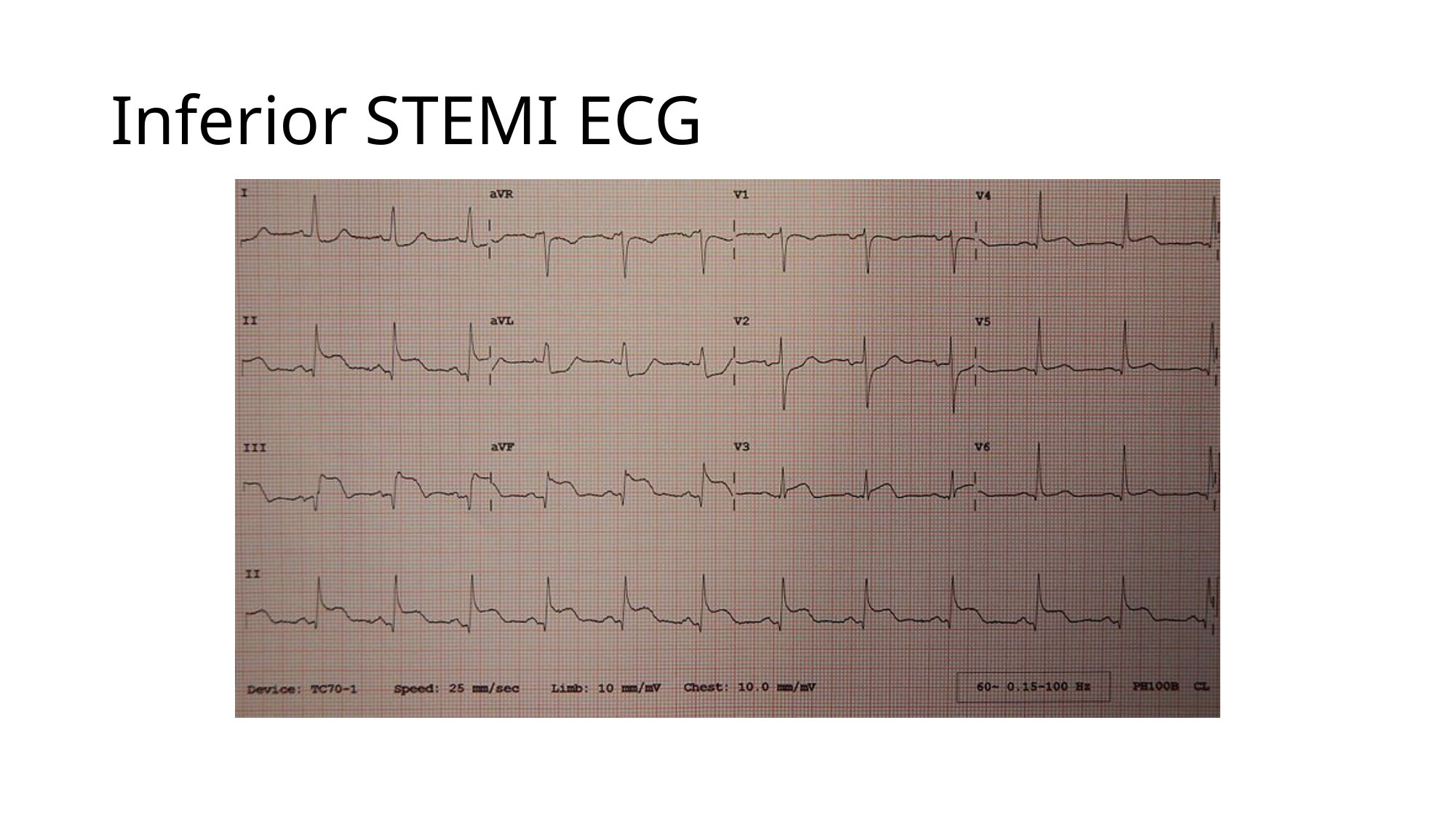

# Inferior STEMI ECG

## Slide 3
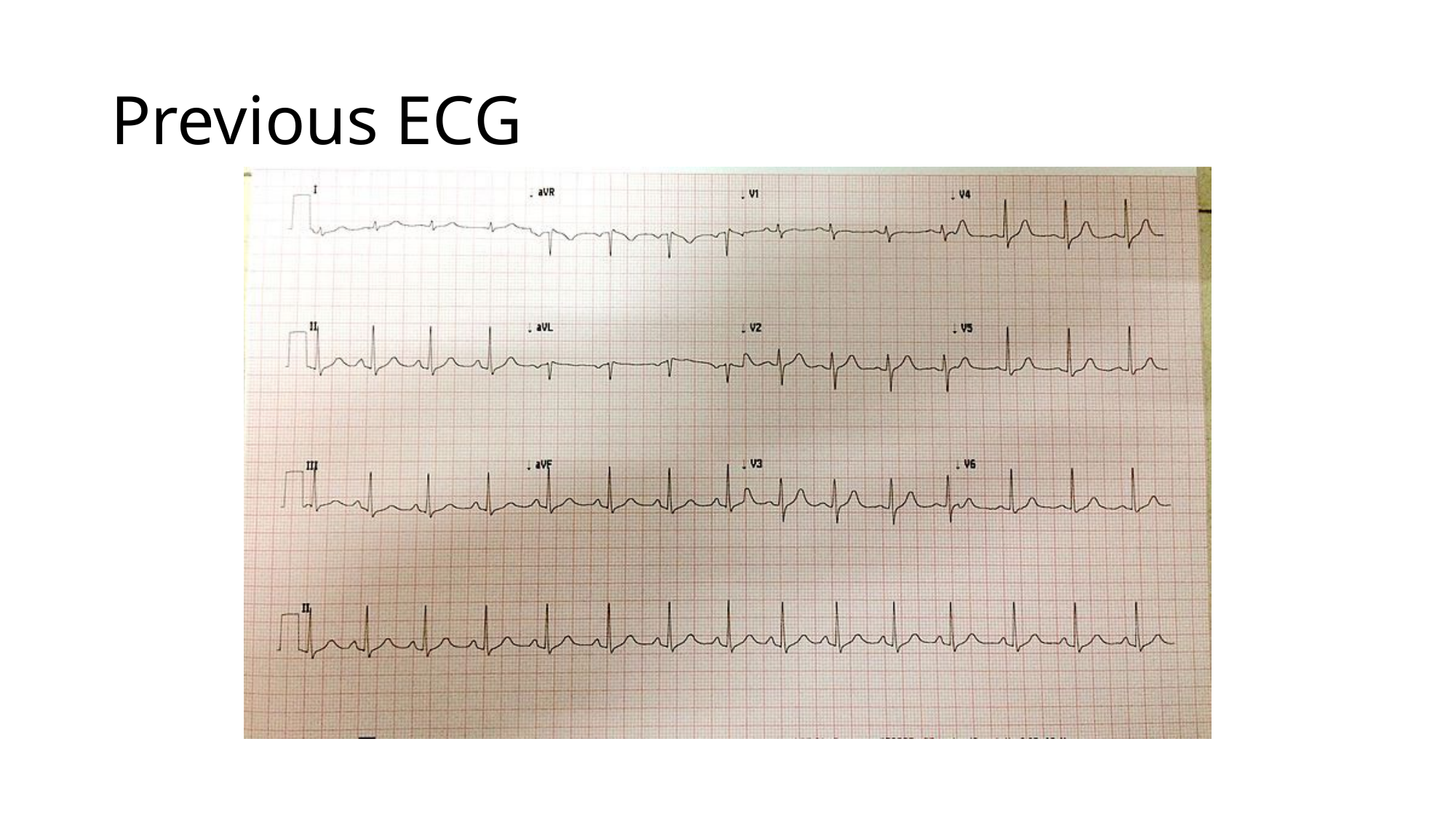

# Previous ECG

## Slide 4
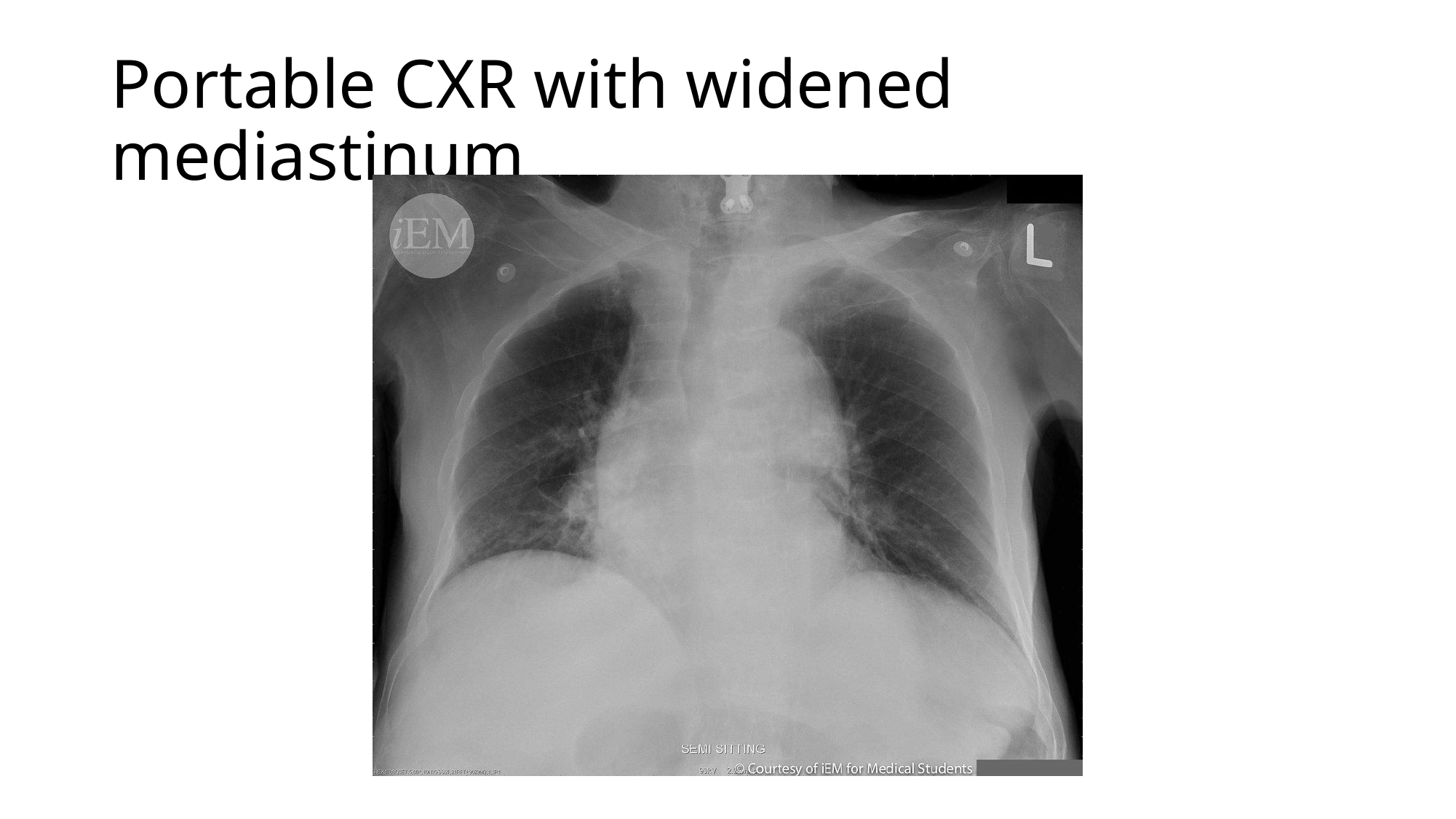

# Portable CXR with widened mediastinum

## Slide 5
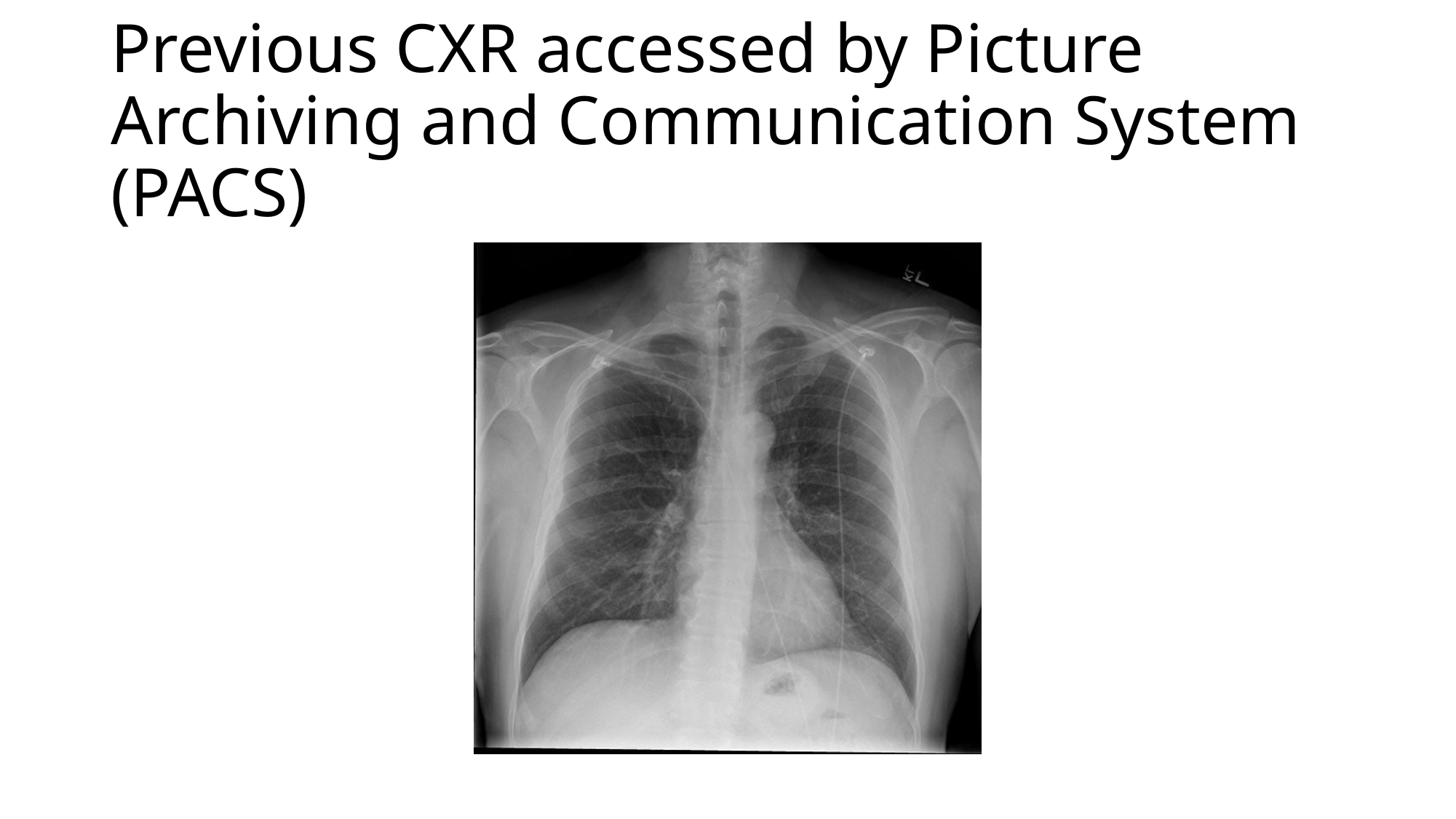

# Previous CXR accessed by Picture Archiving and Communication System (PACS)

## Slide 6
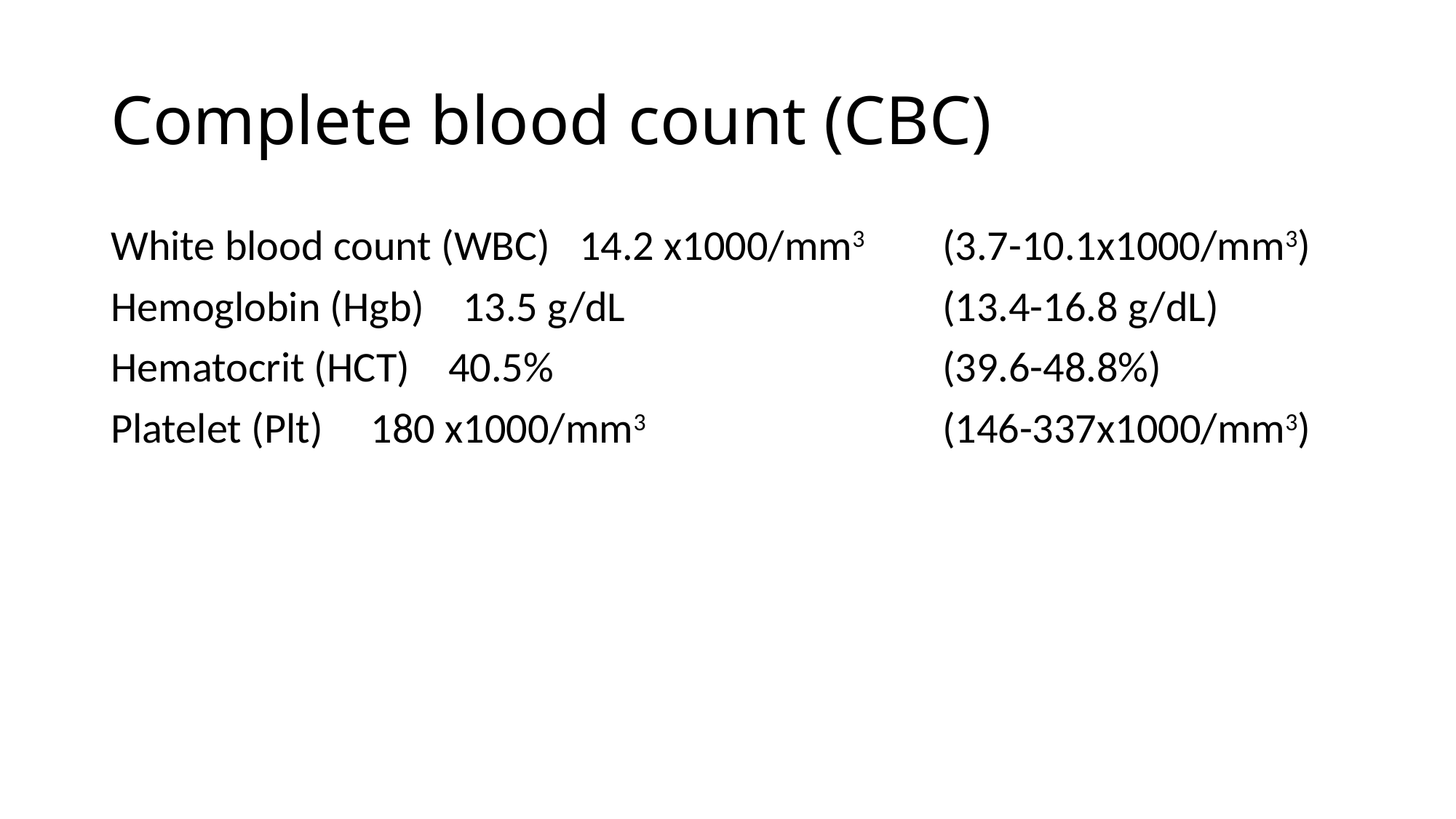

# Complete blood count (CBC)
White blood count (WBC) 14.2 x1000/mm3	 (3.7-10.1x1000/mm3)
Hemoglobin (Hgb) 13.5 g/dL			 (13.4-16.8 g/dL)
Hematocrit (HCT) 40.5% 			 (39.6-48.8%)
Platelet (Plt) 180 x1000/mm3 		 (146-337x1000/mm3)

## Slide 7
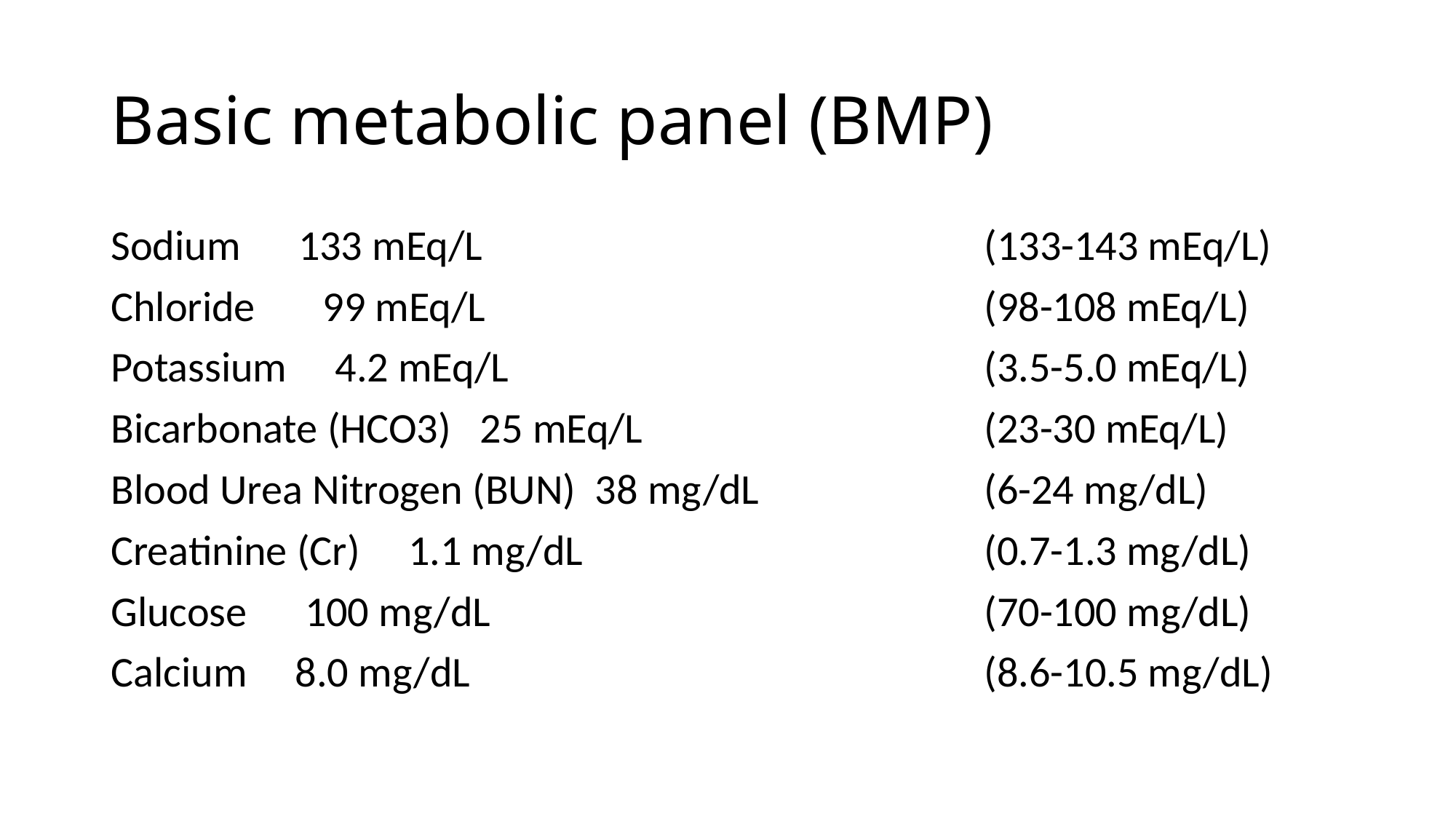

# Basic metabolic panel (BMP)
Sodium 133 mEq/L 					(133-143 mEq/L)
Chloride 99 mEq/L 					(98-108 mEq/L)
Potassium 4.2 mEq/L 					(3.5-5.0 mEq/L)
Bicarbonate (HCO3) 25 mEq/L 			(23-30 mEq/L)
Blood Urea Nitrogen (BUN) 38 mg/dL 		(6-24 mg/dL)
Creatinine (Cr) 1.1 mg/dL 				(0.7-1.3 mg/dL)
Glucose 100 mg/dL 					(70-100 mg/dL)
Calcium 8.0 mg/dL 					(8.6-10.5 mg/dL)

## Slide 8
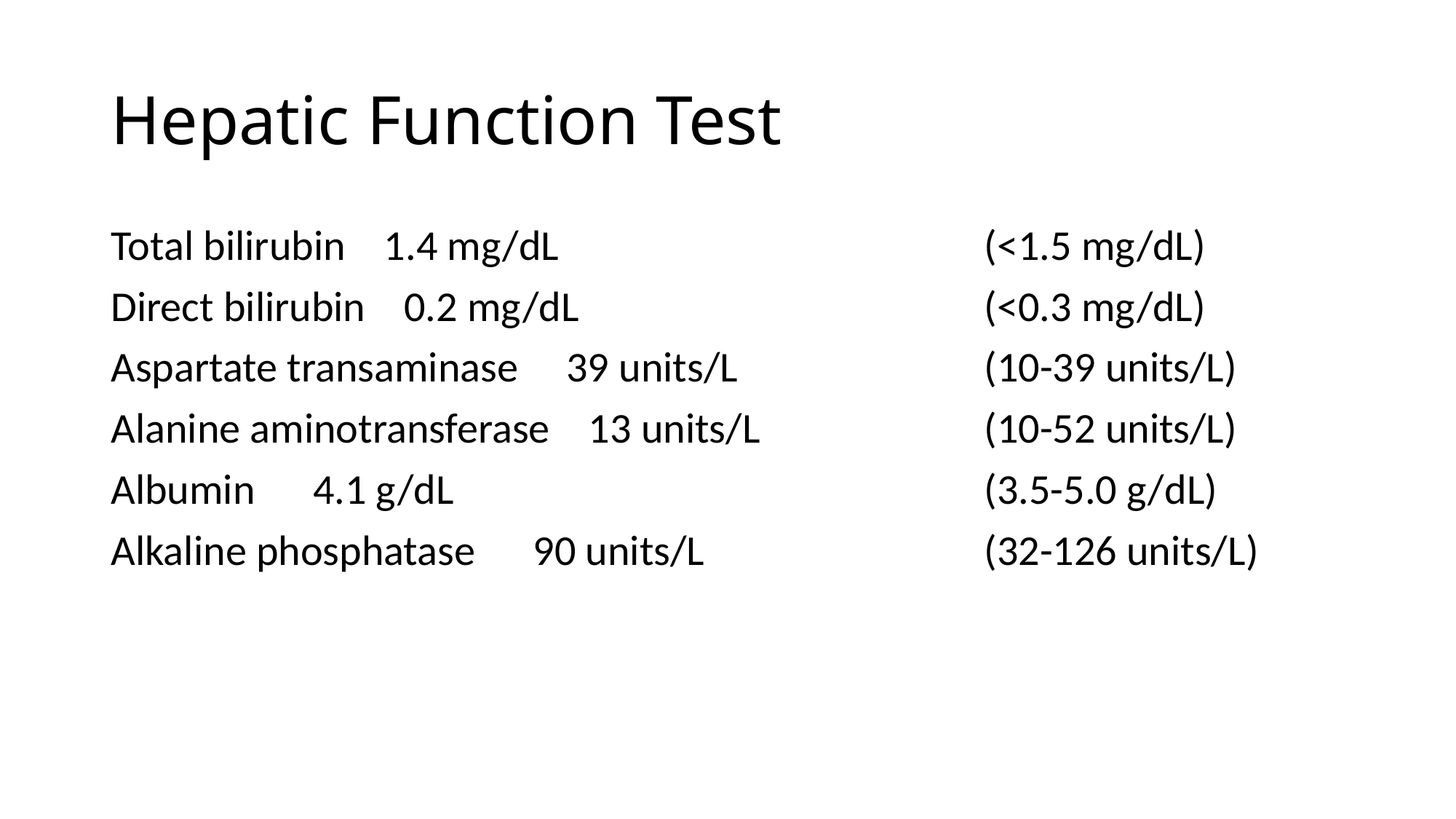

# Hepatic Function Test
Total bilirubin 1.4 mg/dL				(<1.5 mg/dL)
Direct bilirubin 0.2 mg/dL				(<0.3 mg/dL)
Aspartate transaminase 39 units/L			(10-39 units/L)
Alanine aminotransferase 13 units/L			(10-52 units/L)
Albumin 4.1 g/dL					(3.5-5.0 g/dL)
Alkaline phosphatase 90 units/L			(32-126 units/L)

## Slide 9
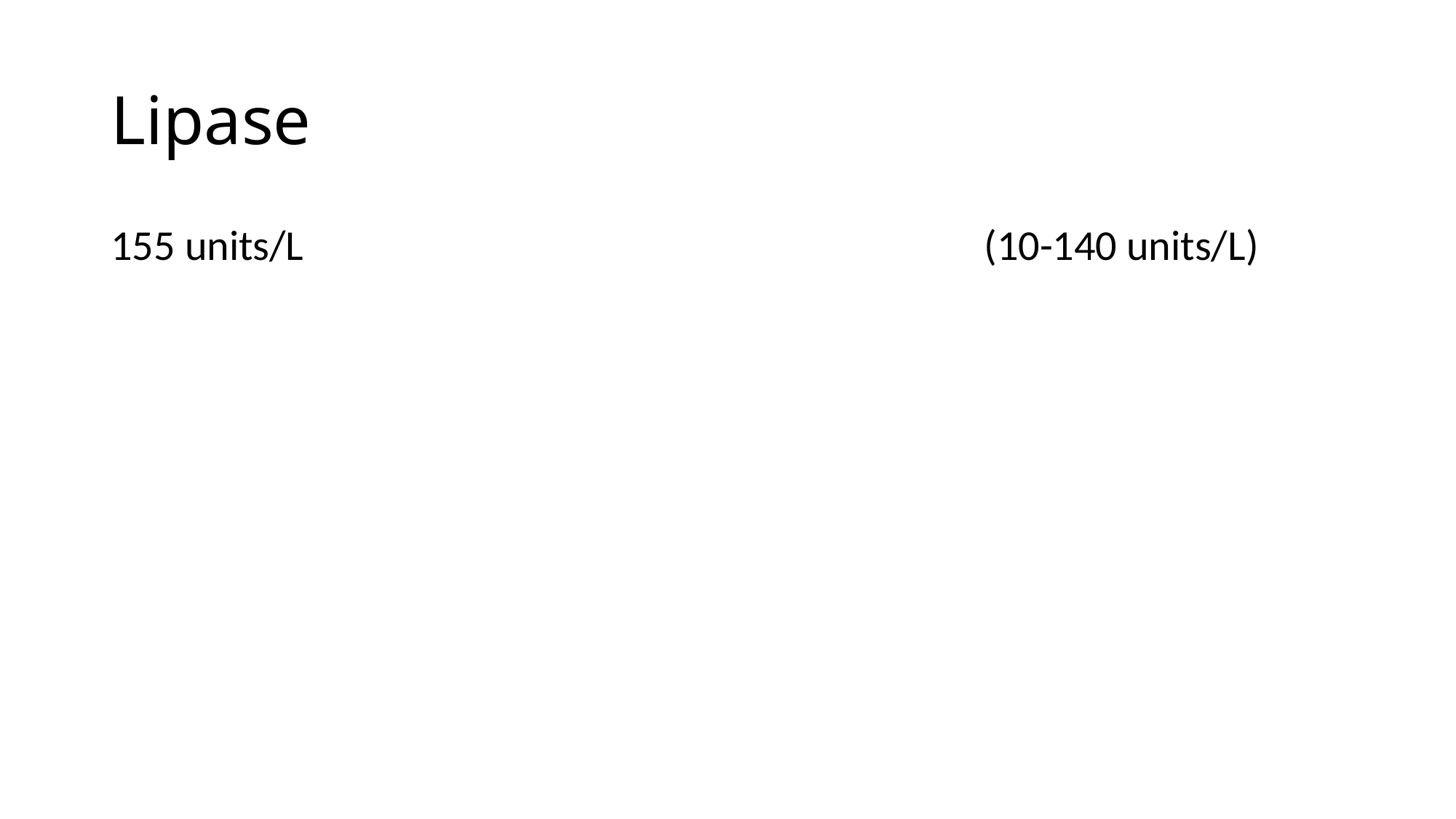

# Lipase
155 units/L							(10-140 units/L)

## Slide 10
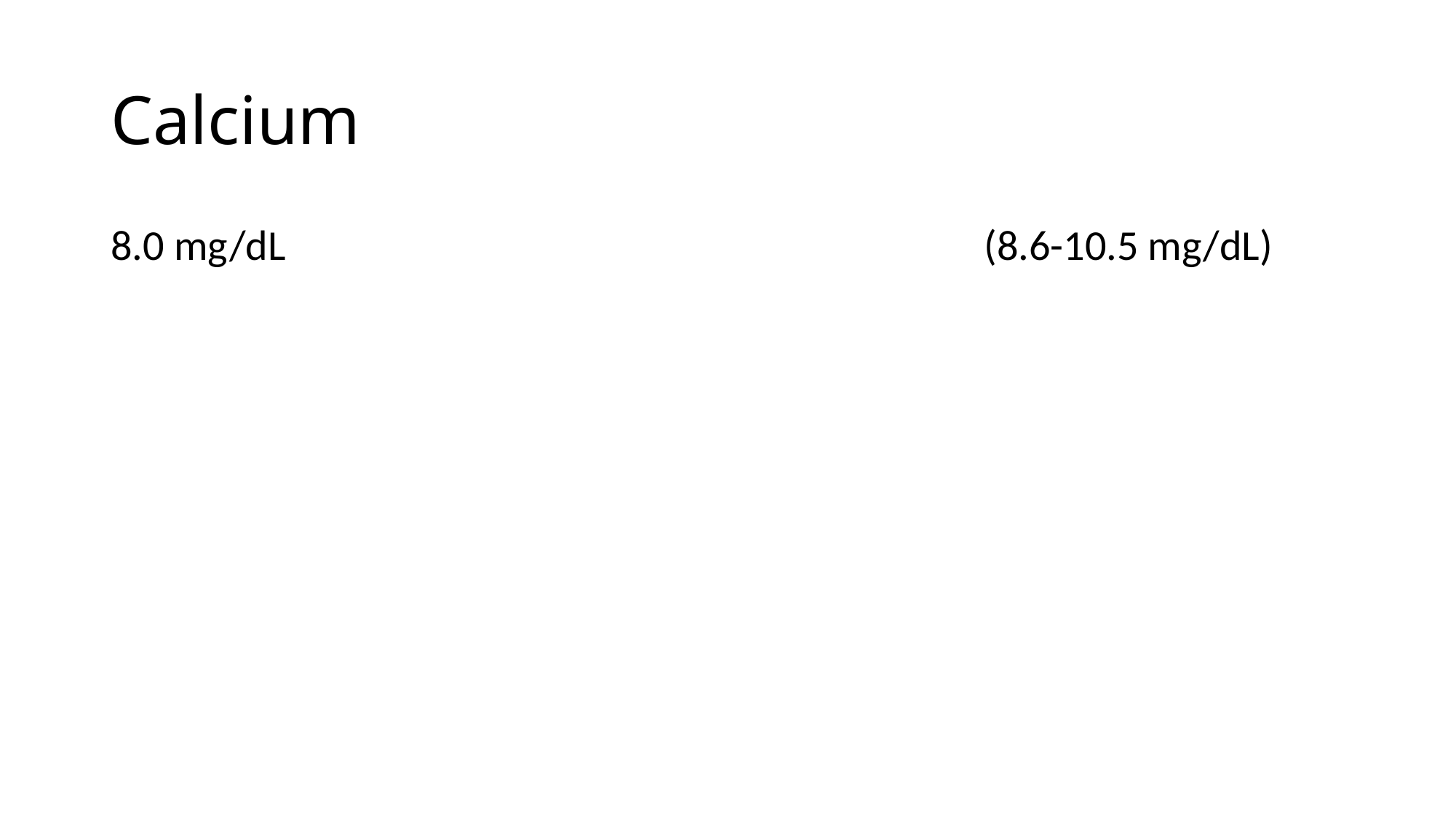

# Calcium
8.0 mg/dL 							(8.6-10.5 mg/dL)

## Slide 11
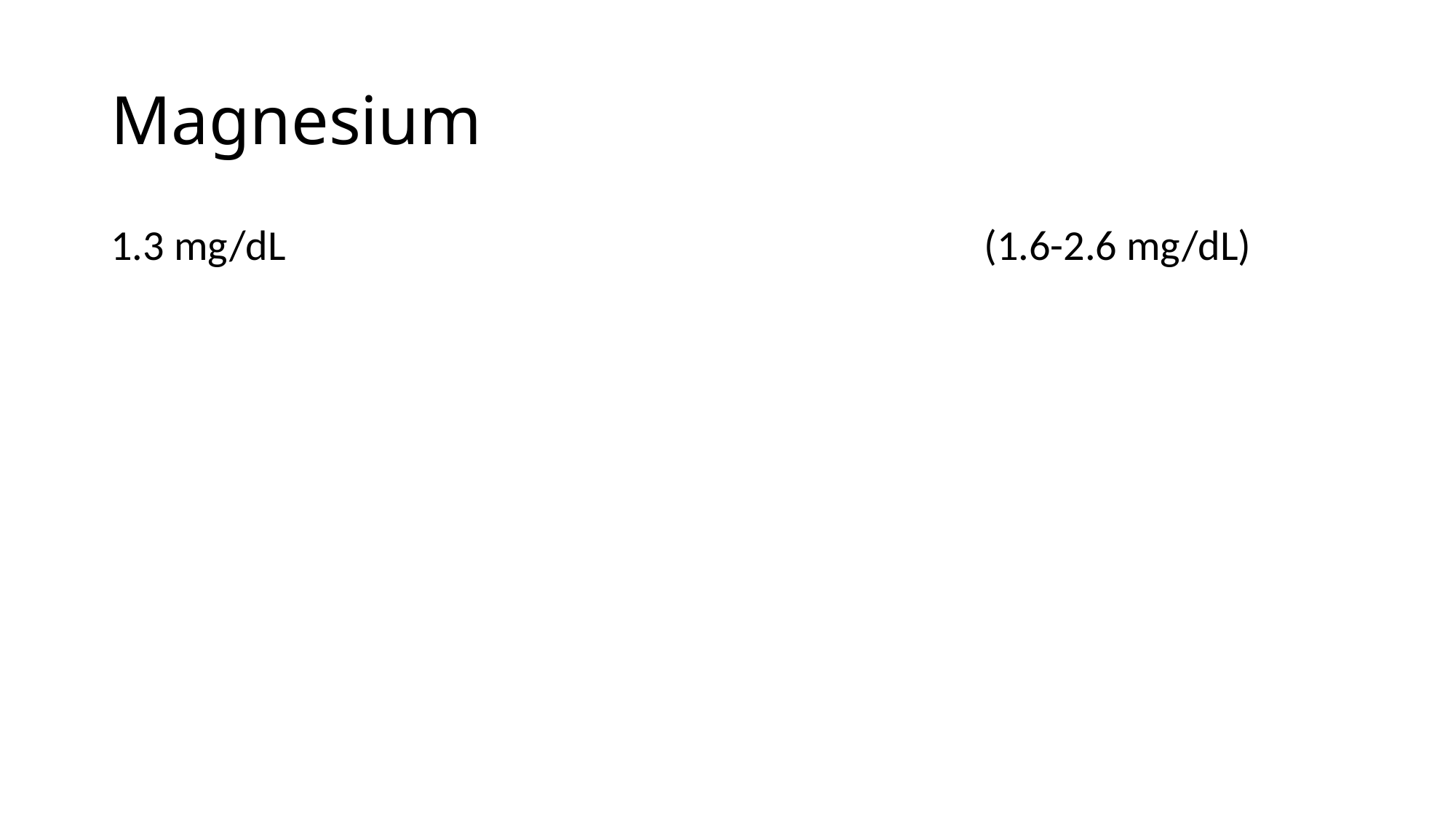

# Magnesium
1.3 mg/dL							(1.6-2.6 mg/dL)

## Slide 12
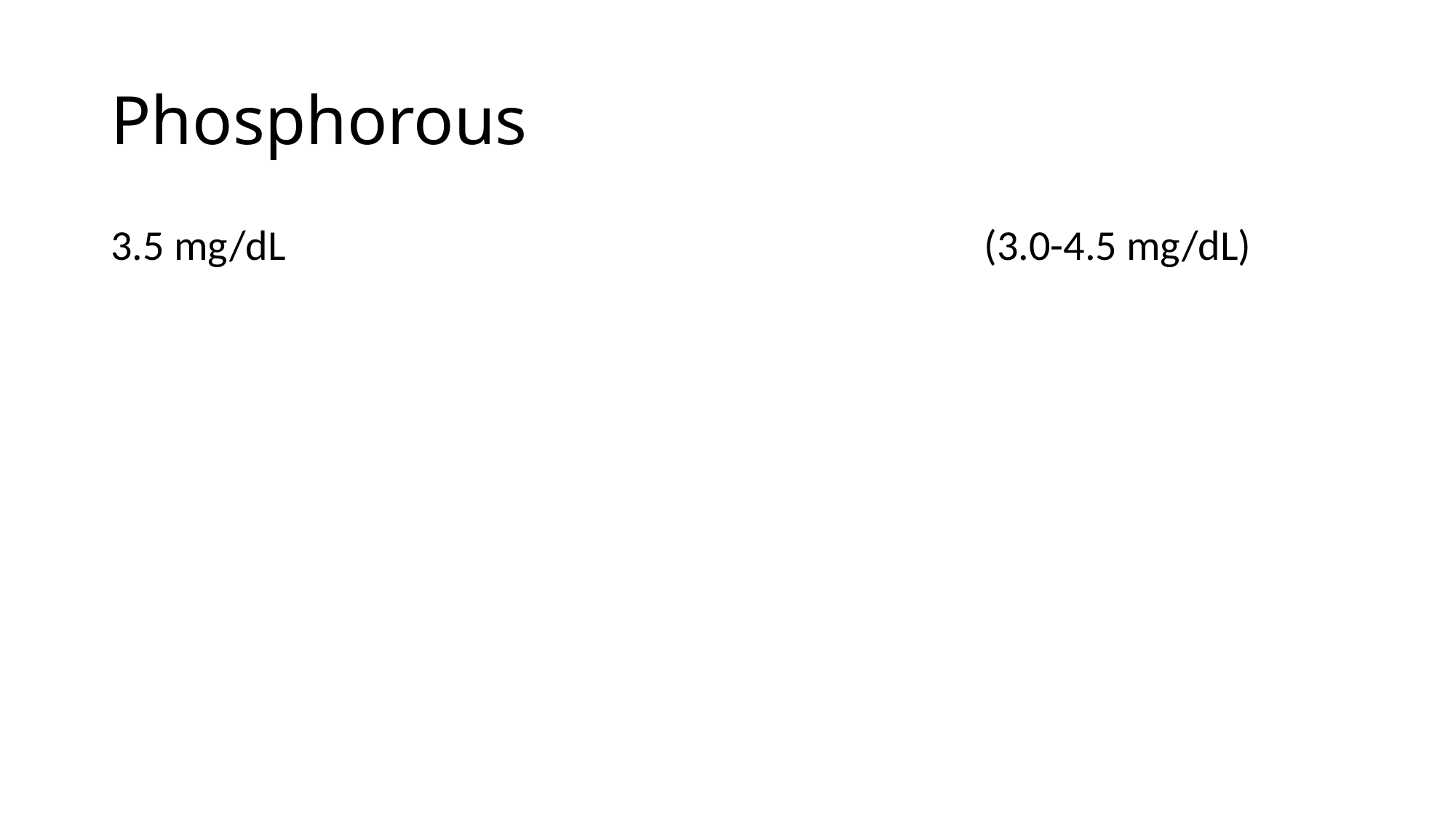

# Phosphorous
3.5 mg/dL							(3.0-4.5 mg/dL)

## Slide 13
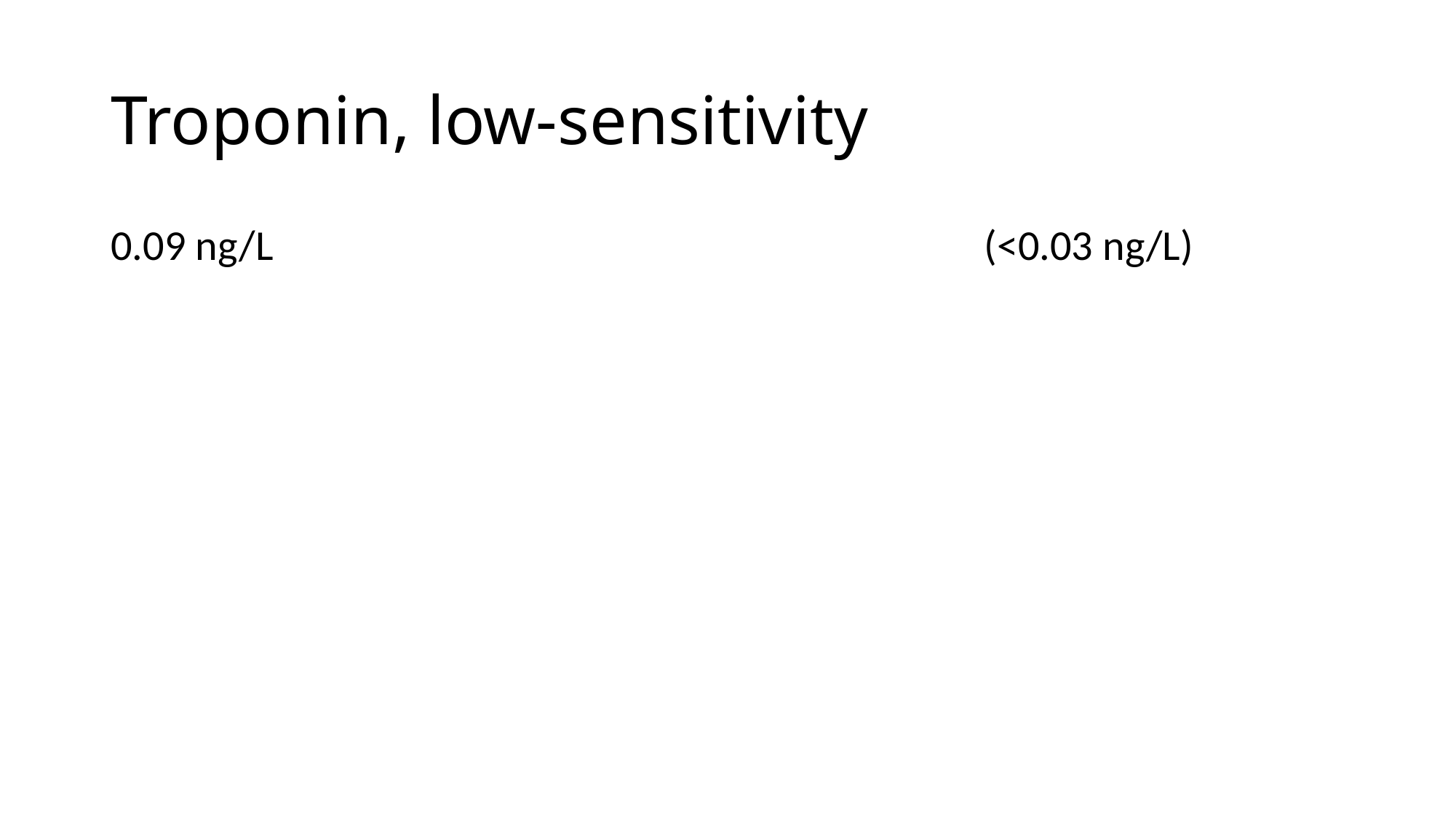

# Troponin, low-sensitivity
0.09 ng/L							(<0.03 ng/L)

## Slide 14
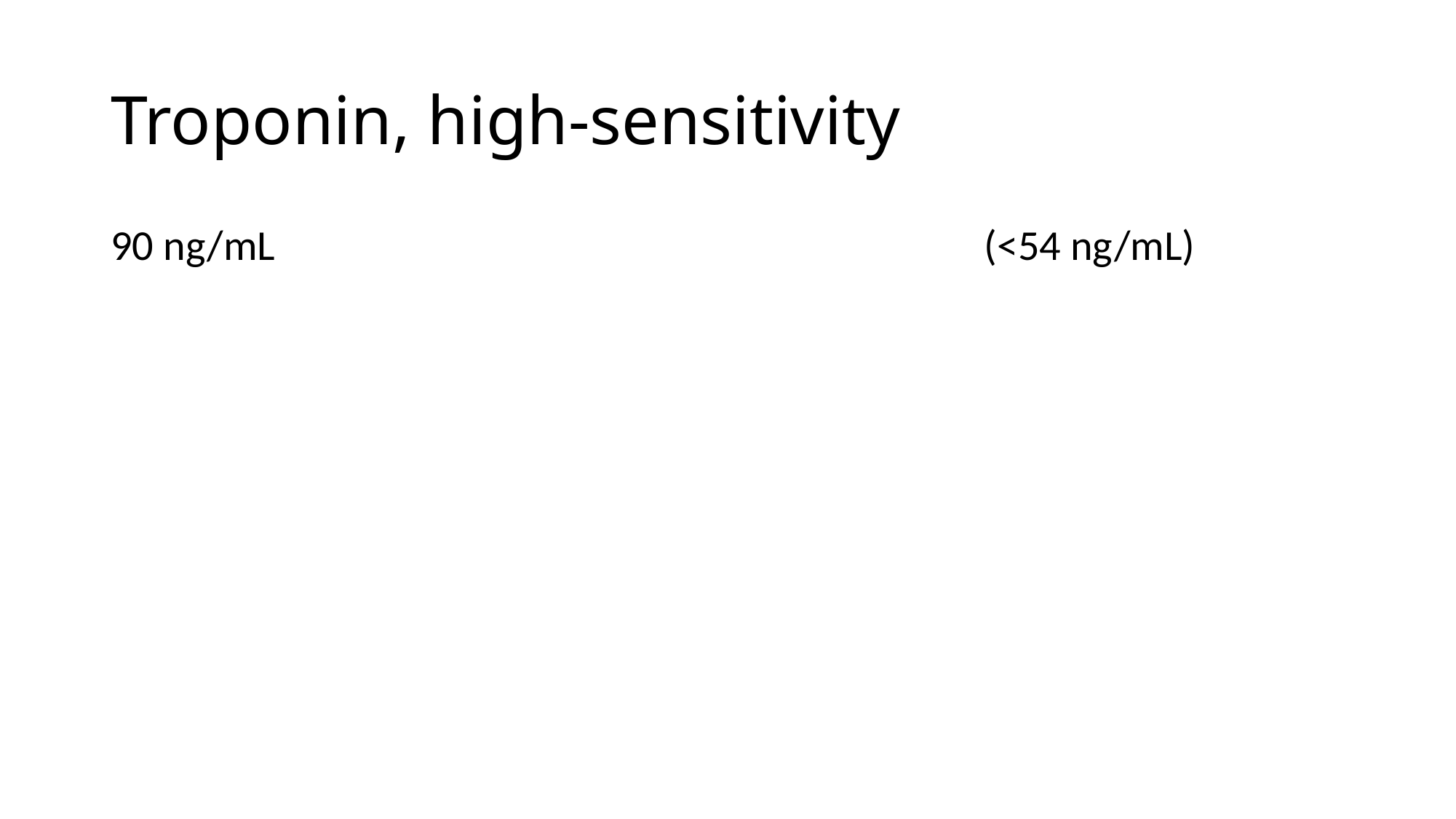

# Troponin, high-sensitivity
90 ng/mL							(<54 ng/mL)

## Slide 15
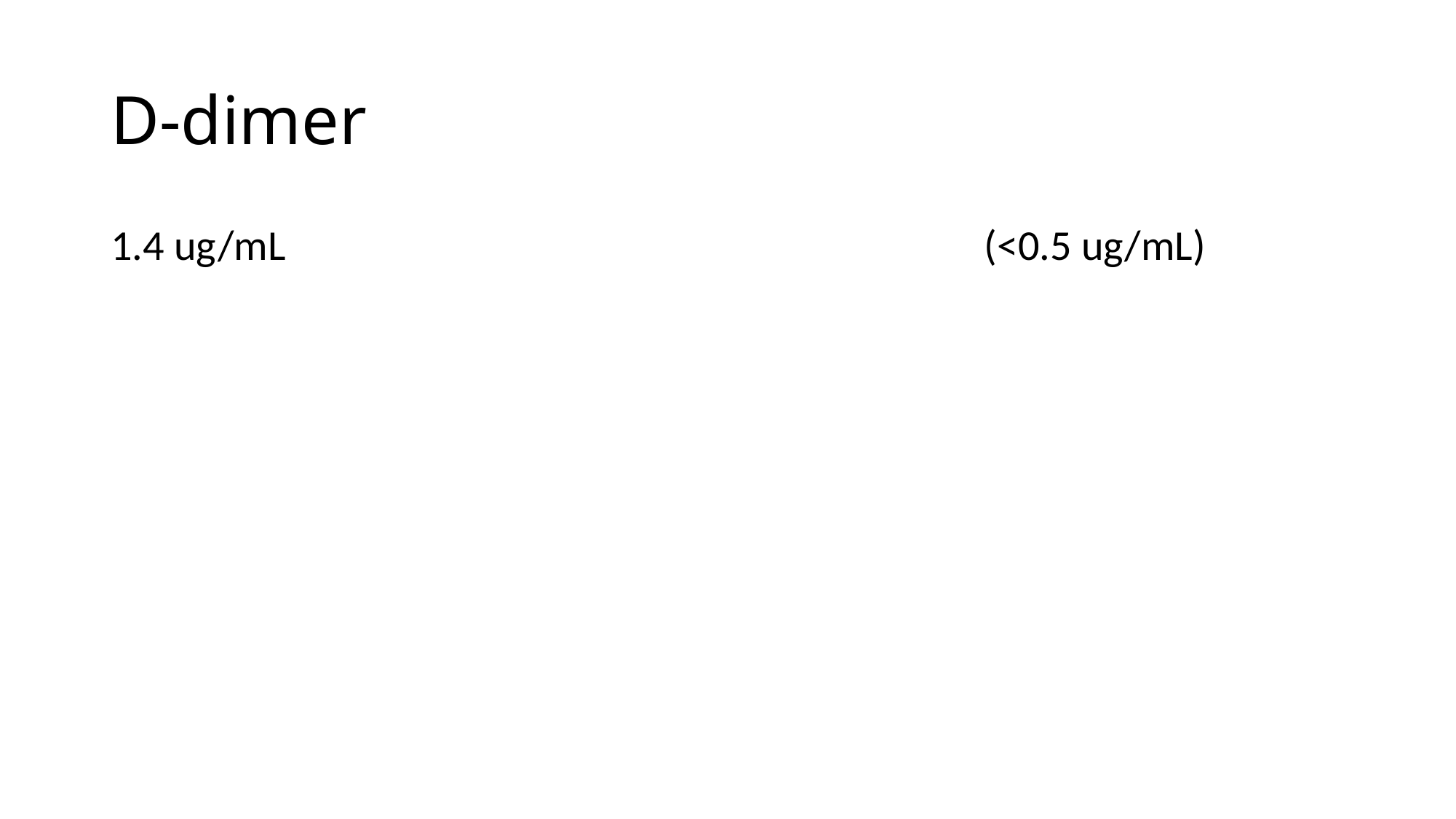

# D-dimer
1.4 ug/mL							(<0.5 ug/mL)

## Slide 16
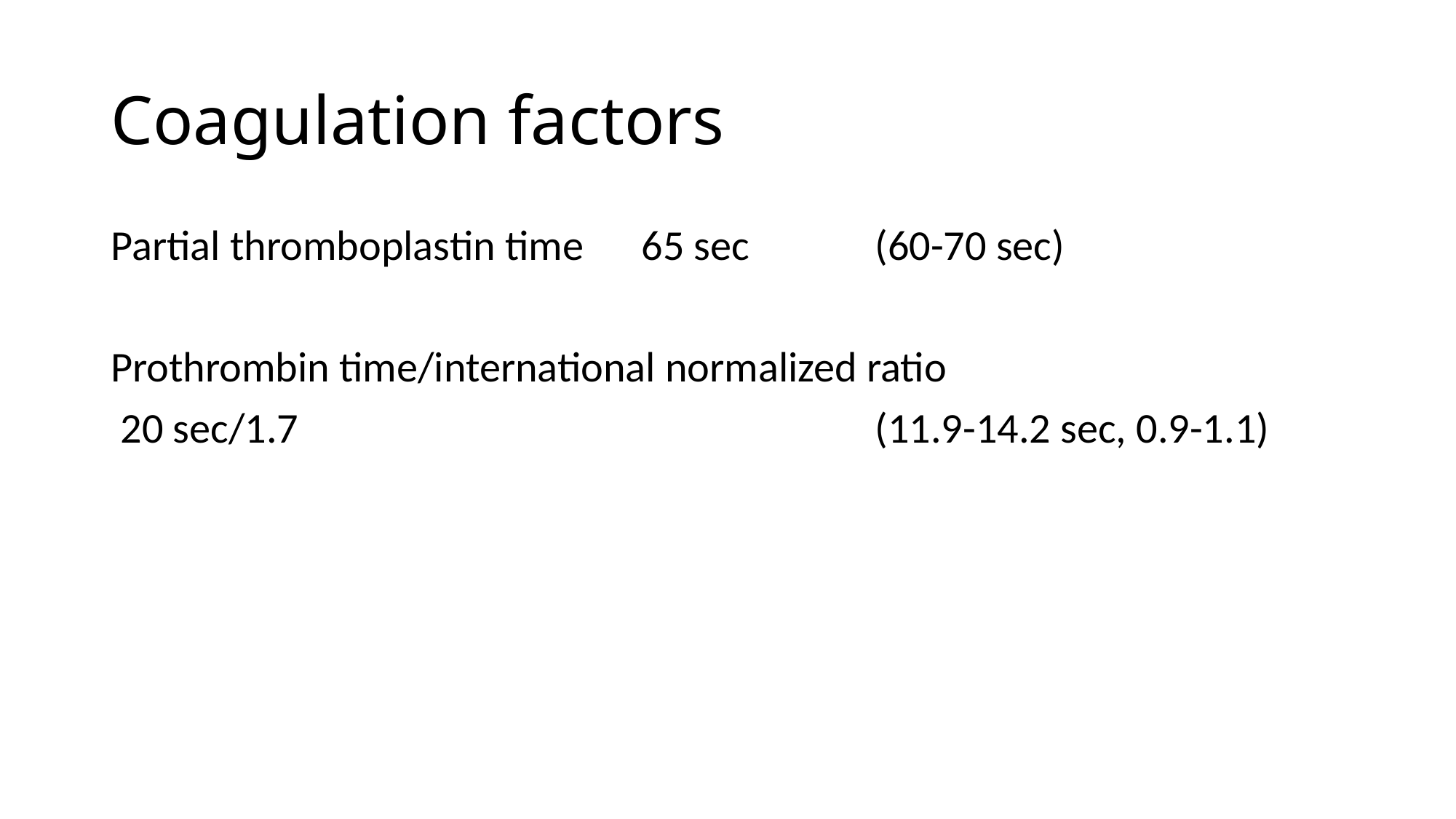

# Coagulation factors
Partial thromboplastin time 65 sec		(60-70 sec)
Prothrombin time/international normalized ratio
 20 sec/1.7						(11.9-14.2 sec, 0.9-1.1)

## Slide 17
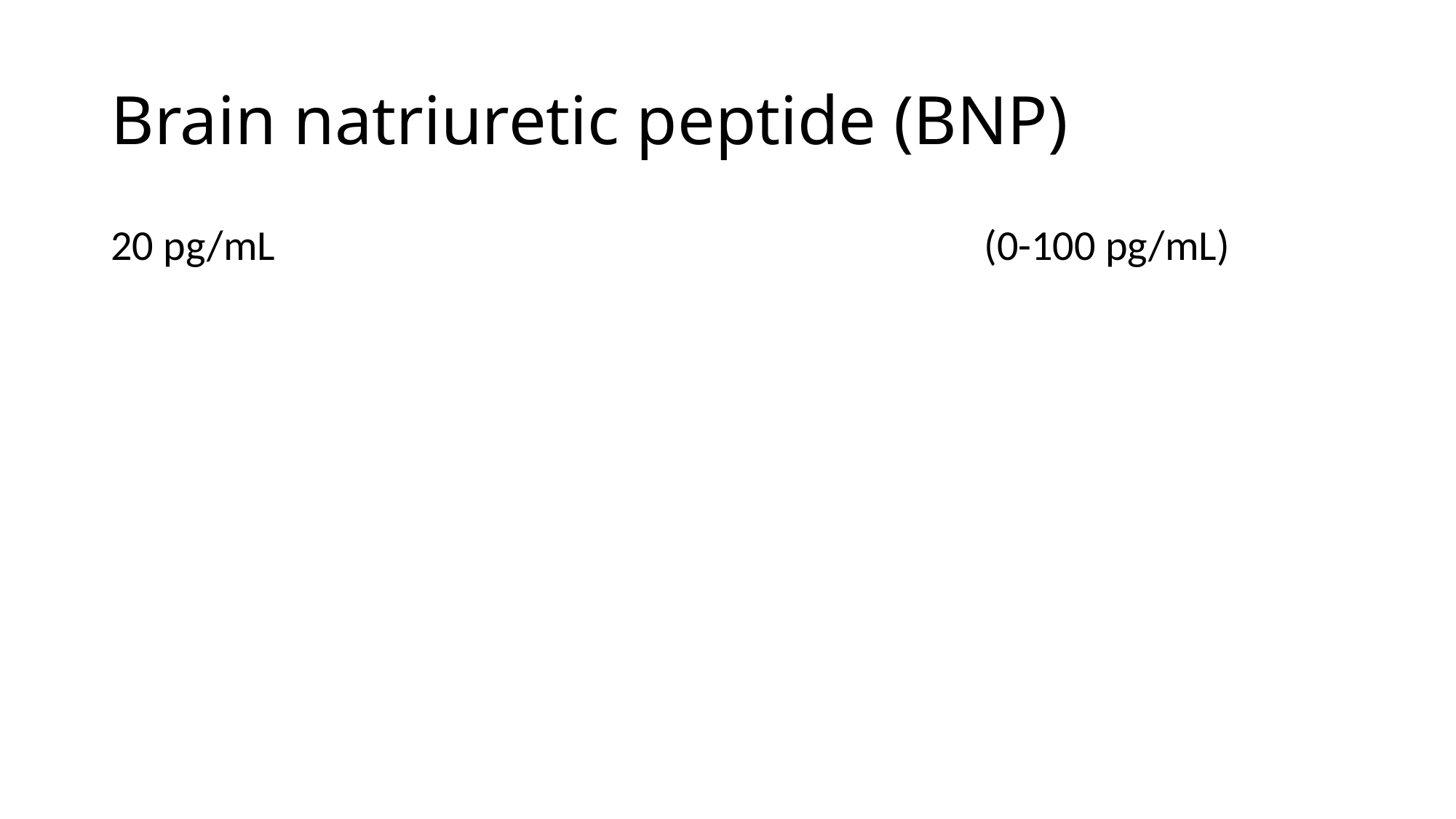

# Brain natriuretic peptide (BNP)
20 pg/mL							(0-100 pg/mL)

## Slide 18
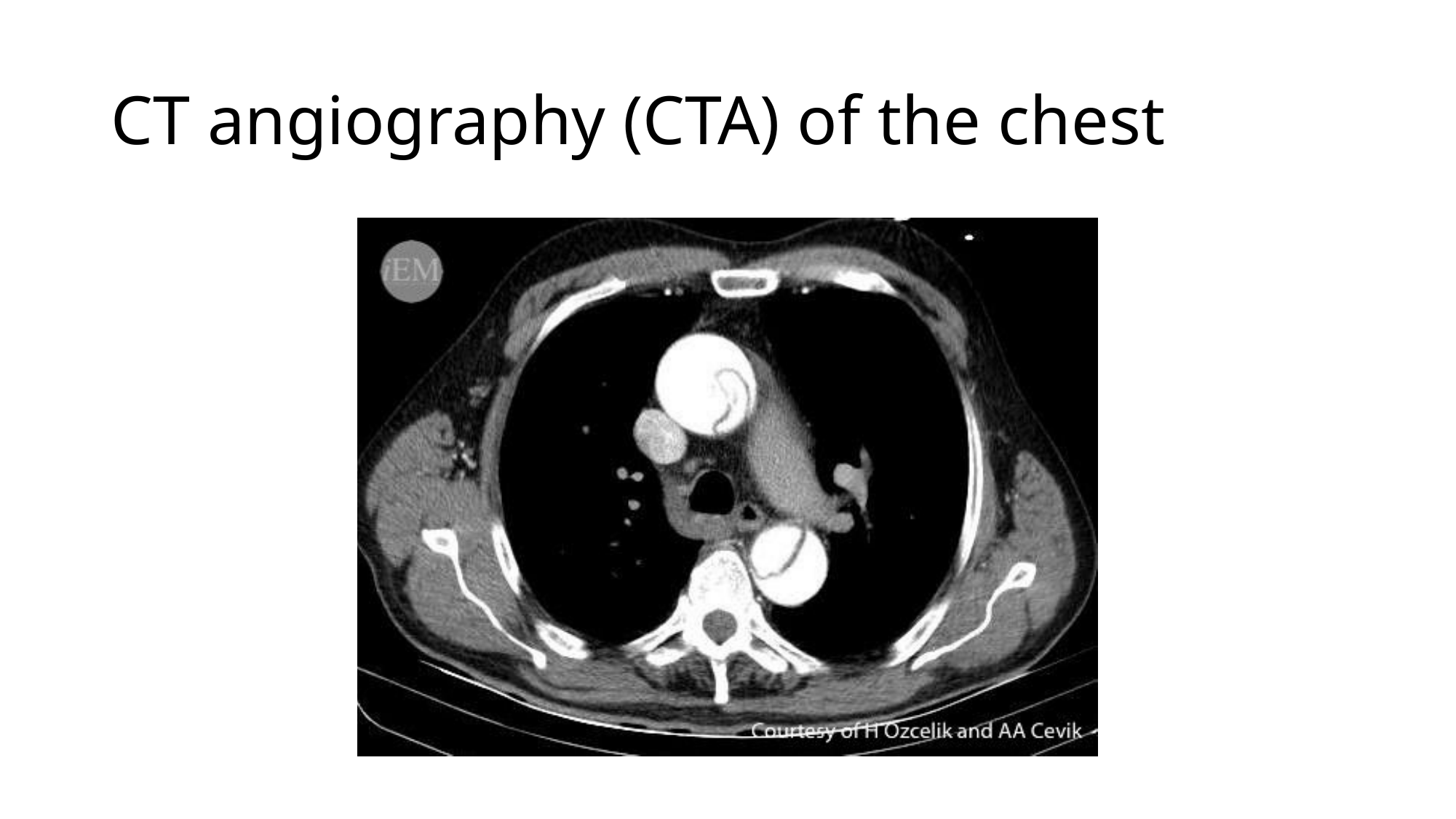

# CT angiography (CTA) of the chest
